# Mitochondrial Phylogenomics of Modern and Ancient Equids

**DOI:** 10.1371/journal.pone.0055950

**Published:** 2013-02-20

**Authors:** Julia T. Vilstrup, Andaine Seguin-Orlando, Mathias Stiller, Aurelien Ginolhac, Maanasa Raghavan, Sandra C. A. Nielsen, Jacobo Weinstock, Duane Froese, Sergei K. Vasiliev, Nikolai D. Ovodov, Joel Clary, Kristofer M. Helgen, Robert C. Fleischer, Alan Cooper, Beth Shapiro, Ludovic Orlando

**Affiliations:** 1 Centre for GeoGenetics, Natural History Museum of Denmark, University of Copenhagen, Copenhagen, Denmark; 2 Department of Ecology and Evolutionary Biology, University of California Santa Cruz, Santa Cruz, California, United States of America; 3 Faculty of Humanities, University of Southampton, Southampton, United Kingdom; 4 Department of Earth and Atmospheric Sciences, University of Alberta, Alberta, Canada; 5 Institute of Archaeology and Ethnography, Russian Academy of Sciences, Novosibirsk, Russia; 6 Laboratory of Archaeology and Paleogeography of Central Siberia, Institute of Archaeology and Ethnography, Russian Academy of Sciences, Novosibirsk, Russia; 7 Centre de Conservation et d’Étude des Collections, Musée des Confluences, Lyon, France; 8 Division of Mammals, National Museum of Natural History, Smithsonian Institution, Washington D.C., United States of America; 9 Center for Conservation and Evolutionary Genetics, Smithsonian National Zoological Park, Smithsonian Institution, Washington D.C., United States of America; 10 Australian Centre for Ancient DNA, School of Earth and Environmental Sciences, The University of Adelaide, South Australia, Australia; Institut de Biologia Evolutiva - Universitat Pompeu Fabra, Spain

## Abstract

The genus *Equus* is richly represented in the fossil record, yet our understanding of taxonomic relationships within this genus remains limited. To estimate the phylogenetic relationships among modern horses, zebras, asses and donkeys, we generated the first data set including complete mitochondrial sequences from all seven extant lineages within the genus *Equus*. Bayesian and Maximum Likelihood phylogenetic inference confirms that zebras are monophyletic within the genus, and the Plains and Grevy’s zebras form a well-supported monophyletic group. Using ancient DNA techniques, we further characterize the complete mitochondrial genomes of three extinct equid lineages (the New World stilt-legged horses, NWSLH; the subgenus *Sussemionus*; and the Quagga, *Equus quagga quagga*). Comparisons with extant taxa confirm the NWSLH as being part of the caballines, and the Quagga and Plains zebras as being conspecific. However, the evolutionary relationships among the non-caballine lineages, including the now-extinct subgenus *Sussemionus,* remain unresolved, most likely due to extremely rapid radiation within this group. The closest living outgroups (rhinos and tapirs) were found to be too phylogenetically distant to calibrate reliable molecular clocks. Additional mitochondrial genome sequence data, including radiocarbon dated ancient equids, will be required before revisiting the exact timing of the lineage radiation leading up to modern equids, which for now were found to have possibly shared a common ancestor as far as up to 4 Million years ago (Mya).

## Introduction

The family Equidae, along with Rhinocerotidae and Tapiridae, is one of the three extant families of odd-toed ungulates (Perissodactyla, Mammalia). The Equidae are richly represented in the fossil record throughout the past 55 Million years (My), starting with dog-sized taxa from the North American Eocene [1]. Equid lineages then spread globally and experienced successive episodes of radiations and extinctions during the early Miocene, the late Miocene, and at the end of the Pleistocene [2], becoming adapted to a variety of environments with remarkable variations in body size [1].

While several dozen extinct equid genera have been described [2], all extant equid species are classified in the same genus, *Equus.* It includes the African wild ass, *Equus africanus,* which is the progenitor of the Domestic donkey, *E. asinus* [3], and three living species of zebra, all endemic to Africa. The zebras include the Plains zebra, also called Burchell’s zebra, *E. quagga* (previously *E. burchellii*); Grevy’s zebra, *E. grevyi*; and the Mountain zebra, *E. zebra* (with two subspecies, *E. z. zebra* of South Africa, and *E. z. hartmannae* of Namibia and Angola) [4]. The genus also includes the Asian wild asses (subgenus *Hemionus*), *E. hemionus* and *E. kiang*, with various recognized subspecies of *E. hemionus* (e.g *E. h. kulan* and *E. h. onager*). Together, the zebras, donkeys, and Asiatic asses make up the non-caballine lineages. The remaining extant lineages, the domesticated horses (*Equus caballus*) and wild populations sometimes referred to as *E. ferus* (Tarpan and/or the Przewalski horse *E. przewalskii*), are classified as caballine horses.

While the phylogenetic relationships among modern equids have received considerable attention in the past few years [3]–[14], no phylogenetic study has been undertaken using complete mitochondrial sequences of all seven extant species of *Equus*. Complete mitochondrial genome (mitogenome) data is currently available for only three species: domestic (142 individuals) and Przewalski (seven individuals) horses, [5]–[7], [15]–[17], one Tibetan Kiang [18], and one domestic donkey [19]. A nearly complete mitochondrial sequence has also been made available recently for one wild donkey individual [6]. Using second-generation sequencing technologies we generate complete mitogenome sequences from 2–4 individuals of all other living equid species. This results in a comprehensive dataset of 14 novel and complete mitogenomes comprising all of the taxonomic diversity of extant equids. Additionally we sequence complete mitogenomes from three extinct equid lineages: the New World Stilt-Legged horses (NWSLH), the *Sussemionus* (*E. ovodovi*), and the Quagga.

NWSLH refer to a group of equids that were endemic to North America. Despite having gracile limbs similar to the Asiatic wild asses, partial mitochondrial DNA sequences revealed no particular genetic affinity with hemiones, instead the NWSLH were nested within caballine horses [20]. Sussemiones (subgenus *Sussemionus*) consist of a lineage of Eurasian equids that survived until the Late Pleistocene, at least up to 46 thousand years ago (Kya) [21], [22]. No particular genetic relationship with any of the living equid lineages was supported by partial mitochondrial sequences [22], and their evolutionary origin is still debated [21], [22]. The Quagga, *E. quagga quagga*, a morphological variant of the Plains zebra found in South Africa, became extinct in the wild in the late 1800 s [23], and is now commonly accepted as a southern variant of the Plains zebra [4], [24].

Inferring the evolutionary relationships among extinct and living equids is an active area of research. So far, analyses have used mainly short fragments of mitochondrial DNA, with highly controversial results suggesting that previously defined lineages should be collapsed into single taxa [20], [22], [25]–[27]. In this study we aim to (1) resolve the phylogenetic relationships among all of the extant as well as three extinct lineages of equids, and (2) to investigate the evolutionary timing of lineage radiation within the genus *Equus*.

## Materials and Methods

### Overall Experimental Design

We isolated one partial and 17 complete mitogenomes representing all extant species within the genus *Equus* (14 modern samples), including three extinct lineages (four ancient samples) (Table 1). For five out of the 14 modern samples, mitogenomes were generated by PCR amplification followed by a combination of Sanger and GS FLX sequencing. The DNA extracts from the remaining nine modern samples and three of the ancient samples were converted into sequencing libraries and shotgun-sequenced using the Illumina HiSeq2000 platform. The mitogenome of the remaining ancient sample (NWSLH sample MS272) was recovered from a genomic library using the MySelect in solution target enrichment kit (MYcroarray, USA) and sequenced on the Illumina HiSeq2000 platform.

**Table 1:** Sample information.

Sample	Origin	Sample type	Name fr. origin	Species	Seq. approach	Amplicons/reads used	Cov.(Cov$)	Length (bp)	Genbank acc. no
JW328	Mineral Hill Cave, NV, USA	Bone	JW328	E. sp. NWSLH	Illumina shotgun	Sh./411	1.8 (1.8)	7,108	JX312726
ACAD 2304	Proskuriakov Cave, Russia	Bone	ACAD2304	E. ovodovi	Illumina shotgun	Sh.+C/18,679	78.8 (79.9)	16,195	JX312734
MS272	Upper Quartz Creek,YT, Canada	Bone	YG 401.268	E. sp. NWSLH	Illumina+capture	C./5,740	35.6 (36.3)	16,074	JX312727
Kulan	Kolmården, Sweden	Whole blood	Kulan	E. hemionus kulan	PCR+FLX seq	6/34,003	791.4 (803)	16,382	JX312728
O91	Reepark, Denmark	Whole blood	Coxys	E. hemionus onager	Illumina shotgun	Sh./6,960	41.7 (42.3)	16,380	JX312730
K41	Tierpark Berlin, Germany	Whole blood	Kiang no.4	E. kiang	Illumina shotgun	Sh./12,474	77.6 (78.8)	16,381	JX312731
K32	Tierpark Berlin, Germany	Whole blood	Kiang no.3	E. kiang	Illumina shotgun	Sh./9,108	55.2 (56.0)	16,382	JX312732
1023	Hobatere, Namibia	Skin	1023	E. zebra hartmannae	PCR+FLX seq	5/35,577	811 (822.9)	16,407	JX312717
1041	Corona (Gamsberg),Namibia	Skin	1041	E. Zebra hartmannae	PCR+FLX seq	6/63,455	1,414.7 (1,435.6)	16,407	JX312718
H11	Tierpark Berlin, Germany	Whole blood	Hartman no.1	E. zebra hartmannae	Illumina shotgun	Sh./6,760	39.8 (40.4)	16,391	JX312724
H21	Tierpark Berlin, Germany	Whole blood	Hartman no.2	E. zebra hartmannae	Illumina shotgun	Sh./11,917	69.6 (70.6)	16,410	JX312719
6390	Buffalo Springs, N/R,Kenya	Skin	6390	E. grevyi	PCR+FLX seq	2/50,554	1,135.7 (1,152.4)	16,403	JX312725
CGG 10096	Longaya Water, Kenya	Dried tissue	USNM 182063	E. grevyi	Illumina shotgun	Sh./1,623	7.5 (7.6)	14,826	JX312720
G51	Aalborg zoo, Denmark	Whole blood	Line (131–2815)	E. grevyi	Illumina shotgun	Sh./13,178	77.8 (78.9)	16,403	JX312722
G42	Aalborg zoo, Denmark	Whole blood	Lise (11D-6CA4)	E. grevyi	Illumina shotgun	Sh./8,128	46.5 (47.1)	16,393	JX312723
6381	L. Nakuru, N.P, Kenya	Skin	6381	E. quagga chapmani	PCR+FLX seq	2/48,010	1,102.9 (1,119.1)	16,408	JX312721
QH1	Musee des Confluences,Lyon, France	Hair	QH1Q1A	E. quagga quagga	Illumina shotgun	Sh.+C/28,573	104.0 (105.5)	16,366	JX312733
CGG 10086	National Zoo, USA	Dried tissue	USNM 259849	E. quagga chapmani	Illumina shotgun	Sh./5,919	31.3 (31.7)	16,251	JX312729

Number of amplicons refers to the number of amplicons sequenced on the GS FLX, and does not include amplicons sequenced by Sanger sequencing. For total number of amplicons see Table S5. Sh. stands for shotgun sequencing and refers to cases where no amplicons have been generated. Sh.+C refers to cases where shotgun and target capture data have been merged. C. refers to in solution MYSelect capture. The number of reads used is the number of reads that mapped successfully to the reference used. Coverage is provided as the total number of bases sequenced and aligned against the horse reference mitogenome divided by the length of the horse reference mitogenome (Cov.) or the horse reference mitogenome after excluding a region of tandem repeats (Cov$). Column mitogenome length provides the total sequence length of the mitogenome that is covered by a minimal read depth of 2 and excluding tandem repeats (see Methods).

### Ethics Statement

Tissue samples were collected by Hans Siegismund [28], or provided by the Smithsonian Institution (Washington D.C.), the Musée des Confluences (Lyon), the Russian Academy of Sciences, the Government of Yukon, or zoological gardens following official agreements with the Natural History Museum of Denmark in the framework of the COSE certificate DK003 of the host institution, which covers CITES-protected species. Permission was obtained from all museums and institutions to access the collections and all samples were on loan for scientific purposes.

### Modern Samples

For PCR amplified mitogenomes, we collected 30–50 mg of hair or dried alcohol-preserved tissue and extracted DNA using the DNeasy Blood and Tissue kit (Qiagen, USA) following the manufacturer’s instructions except that 360 µl of Buffer ATL and 40 µl Proteinase K were used, and samples were left to lyse overnight at 55°C. We performed PCR amplification using three to six overlapping primer sets (Table S5; [16], [17]) as in Vilstrup et al. [29] (Table S4). We pooled purified PCR products from each sample, except for a 1.5 kb fragment, in equimolar amounts and nebulized for 1 min at 2.1 bar to get approximately 600 bp fragments. We then built the fragmented DNA samples into tagged dA-tailed libraries using NEBNext Quick DNA Library Prep Master Mix set for 454 (New England BioLabs, ref: E6090) using a final adaptor concentration of 8 nM. We then pooled the tagged libraries in equimolar amounts and distributed them on one eighth of a plate on the GS FLX. We followed a similar procedure for PCR amplicons from primer sets Pr1 and Pr2, which span the hypervariable region. All tagged reads were sorted, trimmed and mapped to both a donkey and a horse reference genome (Accession Nb. NC001788 and NC001640, respectively) using GS Reference Mapper with default parameters (454 Life Sciences). We allowed for a maximum of one mismatch during index sorting and a minimum size of 80 bp was required during sequence assembly. Consensus sequences were aligned to a reference using Sequencher v4.8 (Genes Code Corporation, Ann Arbor, MI, USA), and the resulting mitogenomes aligned by eye using SE-AL v2.0a11 Carbon (A. Rambaut, Univ. of Oxford). Sanger sequencing of the short 1.5 kb fragment was performed at the Macrogen facility (Seoul, South Korea) with several primers (Table S4).

For shotgun-sequenced mitogenomes, we collected 2 ml blood samples and extracted DNA using the QIAamp DNA Blood Midi kit (Qiagen, USA) following the manufacturer’s protocol except that blood digestion was performed with Proteinase K for 30 min at 70°C, and the final product was eluted into 150 µl EB buffer. We then sonicated the DNA extracts for seven cycles of 15 secs/90 secs (ON/OFF cycles) on mode High (H) using a Bioruptor XL (Diagenode, Belgium). We selected fragments of 200–300 bp using the EZNA Gel Extraction Kit (Omega Bio-Tek, ref: D2500) and converted each sample into an Illumina library using the NEBNext Quick DNA library prep Master mix set for 454 (New England BioLabs, ref: E6090). We amplified the libraries in a 50 µl volume reaction, with two parallel PCR set-ups per library using half (16 µl) of each DNA library. The final PCR reaction consisted of 5U Taq Gold (Invitrogen, Life Technologies), 4 mM MgCl_2_, 1 mg/ml BSA, 1 mM dNTPs, 1 µM of Primer inPE1.0, 20 nM of Primer inPE2.0, and 1 µM of an Multiplexing Index Primer containing a unique 6 nucleotide index tag (Illumina Inc.). PCR cycling conditions consisted of initial denaturation for 10 min at 92°C, followed by 10–15 cycles of 30 secs denaturation at 94°C, 30 secs annealing at 60°C, and 40 secs elongation at 72°C, and a final elongation step at 72°C for 7 min. We QIAquick (Qiagen, USA) purified each amplified library and then quantified them on an Agilent 2100 Bioanalyser. The libraries were then pooled in equimolar ratios, and sequenced in paired-end mode (2×100 bp) on the Illumina HiSeq2000 platform at the Danish National High-throughput Sequencing Centre.

We trimmed the Illumina reads using default settings in AdapterRemoval [30], except using a minimal read length of 25 bp, and aligned reads against both published mitogenomes and those generated here via PCR amplification using BWA [31], disabling the seed and relaxing the edit distance (option –n 0.03) as suggested by Schubert et al. [32]. We removed reads that mapped to multiple positions and with mapping quality scores <25 using SAMtools [31]. We removed sequence duplicates using both read start and read end coordinates for collapsed paired-end reads, and we used only the read start coordinates for filtering potential sequence duplicates for uncollapsed read pairs as implemented in MarkDuplicates from the Picard package (http://picard.sourceforge.net). A final alignment was generated for each mitogenome and visually corrected for potential local misalignments. Finally, we called the sequence of each mitogenome using a consensus approach requiring a minimum base coverage of 2 and over 50% of sequence match among reads.

### Ancient Samples and Museum Specimens

We extracted three ancient samples (JW328, ACAD2304, QH1) and two museum-preserved dried tissue specimens (CGG10086 and CGG10096) at the Centre for GeoGenetics in laboratory facilities dedicated to the analysis of fossil material and geographically separated from laboratories with amplified PCR products. We used three different extraction protocols depending on the tissue type, and all extractions were accompanied by appropriate controls.

We extracted the two bone samples JW328 (20 mg) and sample ACAD2304 (464 mg) following the silica-based protocol described in Orlando et al. [22]. We extracted the two tissue specimens (CGG10086 and CGG10096) as described in Gilbert et al [33], adding 2 mg/mL Proteinase K (New England Biolabs) to the extraction buffer, which we digested overnight at 37°C, followed by QIAquick purification and elution into 50 µl. We extracted ∼50 mg of the hair sample (QH1) as in Gilbert et al. [33] except digestion occurred at room temperature (RT) over three days, after which undigested hair was removed and incubated at RT for an additional three days with more digestion buffer. We concentrated both digests to ∼50 µl using 30 KDa centricons and purified the extracts using QIAquick columns, and eluted the concentrated DNA into 50 µl after a five-minute incubation in elution buffer at RT.

We built indexed Illumina libraries for samples JW328, CGG10086, CGG10096, and QH1 using the NEBNext Quick DNA Library Prep Master Mix set for 454 (New England BioLabs, ref: E6090) following the manufacturer’s instructions, except that SPRI bead purification was replaced by Qiagen MinElute DNA purifications. The libraries were amplified in two steps, as described in Kampmann et al. [34]. We purified the resulting libraries through QIAquick columns, quantified them using an Agilent 2100 Bioanalyser, and pooled them in equimolar ratios for Illumina sequencing performed in single-read mode (75 bp) on an Illumina HiSeq2000 platform at the Danish National High-throughput DNA Sequencing Centre.

We built two independent DNA libraries using 21 µl each of DNA extract for sample ACAD2304 using the NEBNext DNA library prep Master mix set for 454 (New England BioLabs, ref: E6070) and following Meyer and Kircher [35], except working with 25 µl reaction volumes and using a final concentration of 0.5 µM Illumina multiplex blunt-end adaptors. At the end of the final fill-in reaction, *Bst* Polymerase was inactivated following 20 min incubation at 80°C. We then amplified the two libraries for 12 cycles using PCR conditions as described above for the seven modern samples. We purified the PCR products through QIAquick columns and re-amplified them in four parallel reactions (5 µl each) for ten additional cycles. The four amplification products generated per library were QIAquick purified, quantified using an Agilent 2100 Bioanalyser and pooled in equimolar amounts before sequencing in single-read mode (100 bp) on a HiSeq2000 Illumina platform at the Danish National High-throughput DNA Sequencing Centre.

A second Quagga library was also enriched for mitochondrial fragments following the procedure described by Maricic et al. [36] and using 1.5 µg of library as input. Mitochondrial baits consisted of a mix of fragmented, amplified mitogenomes of a Grevy’s zebra, a Somali wild ass, and a Kiang (SF, LF and Primer1 primer sets; Table S4). The captured library was purified through MinElute columns and 16 µl was amplified for 12 cycles in a 50 µl volume under the same PCR conditions as the seven modern samples using 0.2 mM postCap primer inPE1.0 and 0.2 mM post index primer. The captured library was sequenced in single-read mode (75 bp) on an Illumina HiSeq2000 platform at the Danish National High-throughput DNA Sequencing Centre.

We assembled complete mitogenomes following the procedure described for assembling Illumina reads from modern equid specimens, except that for sample JW328 we also attempted to map reads against previously reported partial mitochondrial sequences of hippidiforms (accession numbers: GQ324601, GQ324596 and AY152859) and the complete nucleotide sequence of another NWSLH generated in this study following library target-enrichment (see below). The authenticity of our ancient DNA data was then assessed using the mapDamage package [37] (Figure S3).

The NWSLH sample MS272 was processed separately in a dedicated ancient DNA laboratory at the Pennsylvania State University, USA. We powdered ∼500 mg of bone using a Mikrodismembrator (Braun) and extracted DNA using the column based silica extraction protocol as described in Rohland et al. [38]. DNA was eluted into 60 µl of 1×TET (1×TE including 0.05% Tween20). We prepared an indexed Illumina library using 15 µl of the ancient DNA extract according to the protocol described in Meyer and Kircher [35] with 40 µl reaction volumes. We amplified the library for 25 cycles using a unique indexing primer, and subsequently purified the library using the Agencourt AMPure XP PCR purification kit. We eluted the library in 30 µl of 1×TET as described in Meyer and Kircher [35]. We then enriched the indexed library for the mitogenome using the MYselect (now called MYbaits) target enrichment kit from MYcroarray (USA) following the manufacturer’s instructions. Custom bait molecules were designed based on the modern *Equus caballus* mitogenome sequence (horse genome version equCab2.0 http://genome.ucsc.edu/cgi-bin/hgGateway?db=equCab2 provided by The Broad Institute, Cambridge, USA) at 2X tiling (where each base is covered by two unique bait molecules). To provide enough input DNA for the capture reaction, the library was amplified for another 30 cycles using Phusion Hot Start polymerase (New England BioLabs) and primers IS5_reamp.P5 and IS6_reamp.P7 [35]. The target capture itself was performed to the manufacturer’s specifications. We then amplified the enriched libraries once more using the same Phusion Hot Start polymerase setup including primers IS5_reamp.P5 and IS6_reamp.P7. Following a purification using the Agencourt AMPure XP PCR purification kit, we eluted the final library in 30 µl of 1×TET and visualized the results on a 2% agarose gel.

The post-capture library enriched for the NWSLH mitochondrial DNA was then sequenced as part of a larger pool containing 47 additional libraries at equimolar ratios on one lane of an Illumina HiSeq2000 platform. Raw reads were used for read-merging using Kircher’s MergeReadsFastQ_cc.py script [39], resulting in 8.2 millions of reads that were mapped to the horse genome version equCab2.0 http://genome.ucsc.edu/cgi-bin/hgGateway?db=equCab2 using BWA [31] and SAMtools [40]. The final consensus sequence was called using SAMtools [40] requiring a minimum coverage of 2×.

### Data Partitioning and Model Selection

We included 13 previously published mitogenomes in our analyses (Table S1), and used the online Sequence Manipulation Suite [41] to partition protein-coding genes into first, second, and third codon positions. Two additional partitions were generated: one comprising the two rRNA genes (16S and 12S) and another limited to the control region. A highly variable and repetitive section of the control region consisting of tandem repeats was disregarded (from position 16,125–16,368 on horse ref genome JN398398). Phylogenetic analyses were run independently for each gene as well as on the five partitions data set (15,262 bp), the four partitions data set (14,006 bp; excluding the control region), and on the whole unpartitioned data set. Analyses were run both with and without outgroups consisting of six species of rhinoceros and the lowland tapir (Table S1).

We used jModelTest v0.1.1 [42], [43] and the corrected AIC to select the most appropriate evolutionary model for each gene and each partition. We used seven substitution schemes, base frequencies +F, rate variation +I and +G with eight categories, and ML optimized base tree for likelihood calculations (Table S3a–b). Variable sites and nucleotide composition frequencies were retrieved from MEGA 5 [44].

### Phylogenetic Analyses

Phylogenetic analyses were run using PhyML online [45], [46]. The topology and branch lengths were optimized, and support nodes were estimated using both 500 bootstrap pseudo-replicates and an approximate Likelihood Ratio Test (SH-like) [47]. Topological tests (Approximate Unbiased, AU; and Kishino Hasegawa, KH) were performed using CONSEL v0.3 [48] and site likelihood estimates recovered from PhyML with the –print_site_lnl option. A total of 12 topologies were tested and sorted according to the p-values recovered from AU tests.

RaxML GUI 1.0 [49] was used to compute maximum likelihood analyses on the four and five partitioned data sets using 500 bootstraps. In cases where the best model chosen by jModelTest was not available in RaxML or PhyML the next best model implemented was chosen instead (Table S3a–b). Bayesian phylogenetic analyses were performed using MrBayes v3.1.2 [50]. Two runs of 50 million generations were used for each dataset, and the first 25% of samples from each run was excluded as burn-in.

### Molecular Dating

To place the estimated phylogeny on a calendar time-scale, we ran several additional phylogenetic analyses using the software package BEAST v.1.5.4 [51]. For each analysis, we assumed the relaxed uncorrelated lognormal molecular clock, first with Birth and Deaths, and then with Yule speciation. We ran separate analyses for the four- and five-partition data sets both with and without a non-equid outgroup (see above). MCMC chains were run for 250–500 million generations with 25% burn-in, and with convergence to stationarity determined by visual inspection using Tracer v1.5 [52].

In the analyses including the non-equid outgroup, the molecular clock was calibrated using a normal prior of 55±5 Mya [12], [20], [53] on the root of the Perissodactyl tree. In addition, we ran separate analyses using a second normal prior calibration of 0.7±0.1 Mya for the emergence of the Plains zebra [22] and based on the fossil record of *E. mauritanieus* [54], [55]. In analyses excluding outgroups, only the Plains zebra calibration was used. In the absence of precise carbon dating information, ages for both the NWSLH and the Sussemione were sampled from a uniform prior spanning 12 to 100 Kya.

Monophyly was constrained for species represented in the analysis by more than one individual. The subspecies *E. h. kulan* and *E. h. onager* were not constrained to a single clade. However, following the results of the ML and Bayesian analyses above, we constrained monophyly on the clade comprising all the zebras, the clade comprising all horses (*E. caballus* and *E. przewalskii*), the clade comprising the African asses (*E. africanus somalicus* and *E. asinus*), and the clade containing all equids.

### Branch Tests for Natural Selection

To identify regions of the mitogenome that may be under selection in any given lineage, we performed branch tests for each individual protein-coding gene as implemented in PAML v4.5 [56]. Based on the branch test results, branch-site tests were also run for the NADH dehydrogenase 4 protein gene (ND4) to test the caballine (*E. caballus* and NWSLH) branch for sites under positive selection.

## Results and Discussion

We successfully recovered the complete mitogenome of three extinct equid taxa (NWSLH, Sussemione, and Quagga). We also characterized partial mitochondrial contigs (11–288 bp in length, for a total of 7,108 bp) for a second NWSLH (JW328). Of the modern equids we sequenced 14 full mitogenomes (Table 1). The complete mitogenomes of the NSWLH, Sussemione, *E. hemionus kulan*, and the three species of zebra plus the Quagga had not been sequenced prior to this study. The average depth-of-coverage is 387.3 for modern samples and 57.2 for ancient samples (Table 1).

Minimal pairwise distances were observed between conspecific individuals, suggesting that the sequences reported here are in agreement with the known sequence diversity among equids (Table S8). We evaluated nucleotide misincorporation patterns through comparison of Illumina reads with the horse reference mitogenome following the procedure described in Ginolhac et al. [37]. In contrast to modern samples, typical accumulation of C to T and G to A mismatches were observed at sequence ends in the ancient samples, suggesting that the latter were affected by significant levels of *post-mortem* DNA damage, in particular cytosine deamination (Figure S3). This, together with the base composition of our sequences (Table S6), higher substitution rates at third codon positions over first and second codon positions respectively (data not shown), and the blank experimental controls used during the whole laboratory procedure highlights the quality our sequence data.

### Topological Relationships

**Figure 1:**
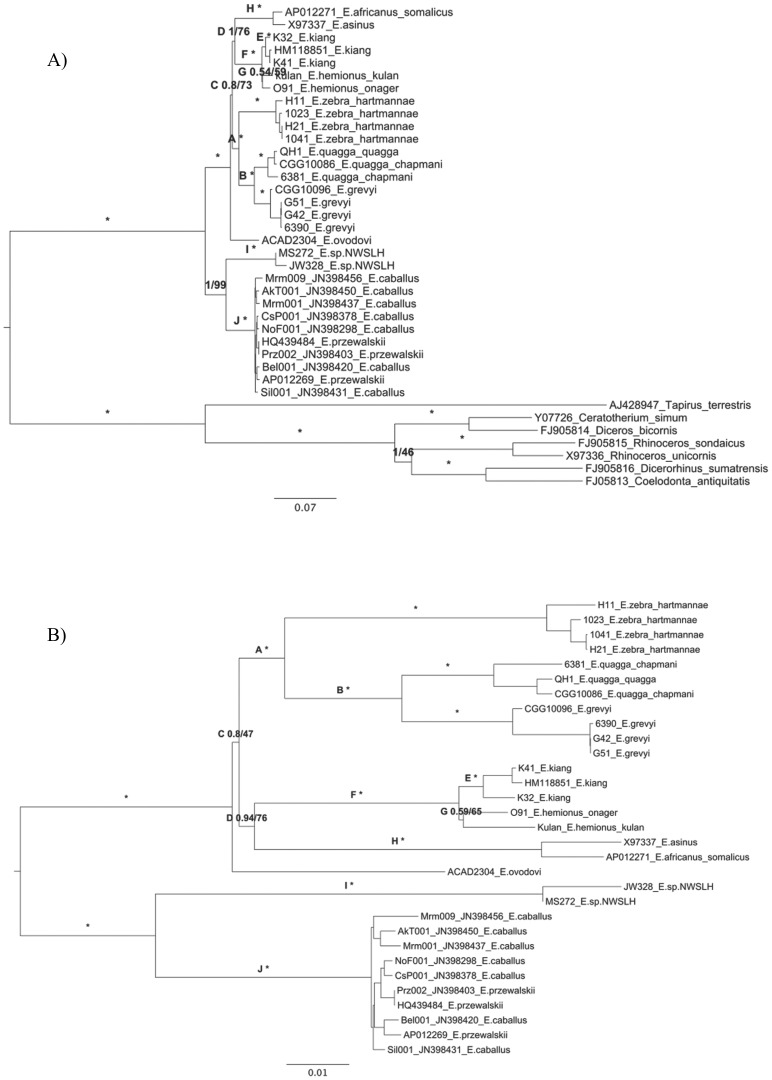
Phylogenetic RAxML trees (GTR+G+I) with 500 bootstraps and MrBayes (GTR+G+I) 50M generations on the full data set. Posterior probabilities are given in proportions and bootstrap support as a percentage on each branch of interest. * Branch is supported by maximum posterior probability and bootstrap (1/100). **A:** Including outgroups and based on 5 partitions. **B:** Excluding outgroups and based on 4 partitions.

Mitogenome alignments were used to perform phylogenetic analyses under a Bayesian and a Maximum Likelihood framework. All methods and sequence partitions considered supported the same topology (Figure 1a–b; Table S2; Figure S2), where all nodes except C, D and G received maximal support (Figure 1a–b). Interestingly, the mitochondrial topology appeared very similar to a study based on 22 partial mitochondrial and nuclear genes [57] and one reconstructed from ca. 55,000 nuclear SNPs [8]. This appears in strong contrast to other recent cases reported among mammals where the phylogenetic signal retrieved from nuclear and mitochondrial genes were in conflict (e.g for brown and polar bears, [58]; for Denisovans and Neanderthals, [59]).

Rooted phylogenetic analyses supported a major division between caballine horses and non-caballine equids, where horses and NWSLH appeared as sister species within caballine horses, in agreement with previous results based on partial mitochondrial sequences [20], [22]. This suggests that Asiatic wild asses and NWSLH did not originate from a single ancestral population with gracile limbs, but instead that similarities in their post-cranial skeleton [25], [60] derive from convergent evolution. However, another possibility is that gracile limbs is the ancestral state which has been preserved in the Asian asses, while it has been lost in all other lineages, resulting in limb states derived from a more gracile one.

Zebras were found to be monophyletic (node A, Figure 1a–b), with the Mountain zebra (*E. zebra*) diverging before the Plains (*E. quagga*) and Grevy’s (*E. grevyi*) zebras, which are sister species. This topology is supported by other studies using a variety of genetic markers [8], [12], [24], [57], [60], [61]. The now extinct Quagga nests within the Plains zebra clade, confirming their conspecific relationship [24]. Asiatic wild asses (node F, Figure 1a–b) can also be confirmed as monophyletic. The subspecies *E. h. onager* and *E. h. kulan* clustered together (albeit with relatively low bootstrap support) in a group divergent from *E. kiang*.

Sussemiones (as represented by *E. ovodovi*) were confirmed as a distinct branch within non-caballine equids (Figure 1a–b; Figure S2), as suggested by previous analyses based on partial mitochondrial sequence information [22]. The Sussemione lineage showed greater genetic distance to caballine horses (Mean = 0.0554±0.0036) than to non-caballine equids (Mean = 0.0427±0.0026) (Table S2; Table S8). Approximately Unbiased and Shimodaira-Hasegawa topological tests showed significantly more support for a topology nesting Sussemiones within non-caballine equids (Figure S2; item 8 in Table S2). However, even though the full length of the mitogenome was characterized, the exact placement of Sussemiones within non-caballines could not be resolved with high confidence (Figure 1a–b, Figure S2; and topological tests presented in Table S2), suggesting that additional phylogenetic information from nuclear genes and/or the identification of e.g rare genomic rearrangements or insertion sites of transposable elements will be required for unraveling the phylogenetic origin of this enigmatic equid lineage [21], [62].

In Bayesian analyses, African wild asses and domestic donkeys were supported as a sister lineage to Asiatic wild asses, a pattern compatible with various past suggestions that the subgenus *Asinus* should incorporate both African and Asiatic asses; however this relationship was not mirrored in ML analyses (node D, Figure 1a–b). Therefore, the mitochondrial information should be regarded as inconclusive with regards to the relationships between the main non-caballine groups (Sussemiones, Asiatic wild asses, African wild asses and donkeys, and zebras). Interestingly, SNP chip data comparing >40K autosomal markers [8] and a study combining nuclear and mitochondrial genes [57] could not resolve these relationships with high support either. This suggests that non-caballine equids have likely experienced an extremely rapid radiation. Alternatively, incomplete lineage sorting within the non-caballine ancestral population might explain the persistence of donkey-like mitochondrial sequences in the Asiatic wild ass lineage, resulting in spurious phylogenetic reconstructions.

The emergence of new approaches using NGS, including whole exome capture, or even complete genome characterization, will soon be available to determine whether the non-caballine phylogeny corresponds to a hard or soft polytomy [63], [64], or incomplete lineage sorting [65]. For now, we note that many other studies have also found a sister relationship between African wild asses and Asiatic wild asses [8], [57], [66]–[69] (but see [12]).

### Branch Tests for Natural Selection

We used the PAML package [56] to estimate non-synonymous to synonymous rate ratios (dN/dS) along the main branches of the topology (Figure S1) and compared this model to a null model that assumes one conserved ratio across the whole phylogeny (Table 2). For all genes except *COX2*, *ND2*, *ND4* and *ND5*, the null model was better supported and dN/dS ratios were found to be significantly lower than 1 (Table S7), in agreement with the presence of purifying selection acting on mitochondrial genes. For *ND4* (NADH dehydrogenase 4), the dN/dS ratio of the ancestral branch leading to caballine horses (horses and NWSLH) was found to be significantly superior to 1 (Table S7), suggesting positive selection for non-synonymous variation during the early evolutionary history of that lineage. In order to identify which amino acid(s) could have been functionally advantageous, we performed branch-site tests in the branch ancestral to caballine horses compared to the rest of the topology for gene *ND4*. However, this analysis did not identify any amino acids that were supported as having evolved under positive selection (p-value: >0.25).

**Table 2:** Average node ages from BEAST.

Node	With outgroup	Without outgroup
Root	5.43E+07 (5.37E+07–5.47E+07)	N/a
Plains zebra	1.06E+06 (7.09E+05–1.87E+06)	6.86E+05 (6.852E+05–6.858E+05)
Grevy’s+Plains (B)	2.80E+06 (1.90E+06–3.60E+06)	1.46E+06 (1.42E+06–1.51E+06)
Grevy’s zebra	7.41E+05 (5.17E+05–1.1E+06)	3.93E+05 (3.84E+05–4.01E+05)
Horses (J)	6.37E+05 (4.13E+05–8.45E+05)	3.64E+05 (3.36E+05–3.89E+05)
Horses+NWSLH	5.91E+06 (4.31E+06–8.56E+06)	2.59E+06 (2.39E+06–2.89E+06)
Mountain zebra	8.86E+05 (5.81E+05–1.1E+06)	4.84E+05 (4.66E+05–4.99E+05)
Rhino+Tapir	4.40E+07 (4.24E+07–4.65E+07)	N/a
Rhinos	2.08E+07 (1.7E+07–2.6E+07)	N/a
Sum.+Wool.	1.01E+07 (8.43E+06–1.24E+07)	N/a
Donkey (H)	1.24E+06 (3.19E+05–2.10E+06)	7.13E+05 (7.06E+05–7.23E+05)
Equids	9.38E+06 (6.72E+06–11.86E+06)	4.27E+06 (3.97E+06–4.73E+06)
Non-caballines	5.91E+06 (4.41E+06–7.54E+06)	2.92E+06 (2.81E+06–3.11E+06)
Kiang (E)	6.91E+05 (4.67E+05–1.08E+06)	3.69E+05 (3.55E+05–3.80E+05)
Kulan/onager (G)	1.11E+06 (7.46E+05–1.68E+06)	5.94E+05 (5.67E+05–6.20E+05)
Asses (F)	1.24E+06 (8.29E+05–1.59E+06)	6.72E+05 (6.49E+05–6.88E+05)
Sussemione	6.39E+06 (3.86E+06–9.37E+06)	2.92E+06 (2.79E+06–3.10E+06)
Zebras (A)	4.74E+06 (3.33E+06–6.05E+06)	1.75E+06 (6.65E+05–2.49E+06)
Zeb+Don+Ass (C)	5.92E+06 (3.75E+06–8.75E+06)	2.87E+06 (2.75E+06–3.05E+06)
Donkey+Ass (D)	5.42E+06 (3.43E+06–7.89E+06)	2.62E+06 (2.49E+06–2.80E+06)

Analyses run with and without outgroups (see Table S9a–b). All dates are in years with 95% confidence interval given in parentheses. N/a = not applicable. Sum = Sumatran rhino; Wool = woolly rhino; Zeb = zebras; Don = Donkey; Donkey = *E. africanus* and *E. asinus*; Asses = *E. hemionus* and *E. kiang*; Ass = Asses. Node letters in parentheses as in Figure 1.

### Divergence Times

**Figure 2:**
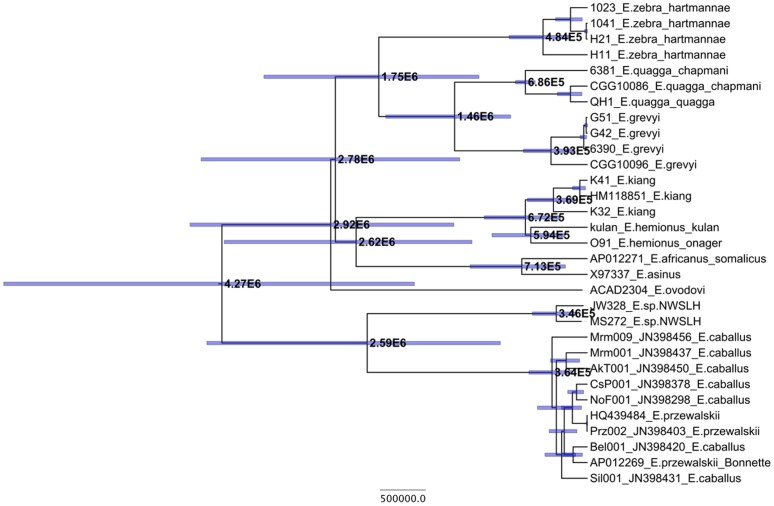
Node ages as estimated from BEAST analyses given in years and with 95% HPD. Shown is the averaged results from the three analyses excluding outgroups (Table 2).

We used relaxed molecular clock (log-uncorrelated) methods as implemented in BEAST [51] to date recent lineage divergence events along the evolutionary history of equids. A total of eleven analyses were run including different data set partitions (4-way or 5-way partitioned), different calibration points (the origin of Perissodactyla at 55 Mya, and/or a recent emergence of the Plains zebra lineage at 0.7 Mya), and different models of speciation (Yule *versus* Birth/Death). The resulting average estimated ages are displayed in Figure 2, Table 2 and Table S9a–b.

The estimated mitochondrial TMRCA for the Equidae varied considerably between our different analyses. When the two outgroup taxa are excluded from the analysis and only the more recent calibration point is used, we estimate the mitochondrial TMRCA of all equids around 4.3 Mya (95% CI: 4.0–4.7 Mya). When the outgroup taxa are included, the average mitochondrial TMRCA increases to 8.6 Mya (95% CI: 6.7–11.9 Mya) (see Table S9a–b). The younger TMRCA is similar to previously reported divergence estimates for the Equidae (3.8 Mya: [12]; 3.9 Mya: [60]; 4.0 Mya: [22]), however ages bracketing that range have also been proposed (2.3 Mya: [10]; 5.8 Mya: [20]). We believe the older age estimates likely result from the contrast between the deep phylogenetic distance separating equids and other living perissodactyls, and the relatively young origin of equids as a whole. Evolutionary rate estimates have been shown to vary in a time-dependent manner [70], [71], with deep calibrations leading to slower evolutionary rate estimates. The posterior distribution of mutation rates recovered from our analyses show this trend (Table 3). This may be due to a variety of effects, including substitution saturation, purifying selection, and model mis-specification, especially with regard to how rate heterogeneity across sites is compensated [72], [73]. The older calibration may therefore be less appropriate for estimating recent divergence times, such as those we aim to address here.

**Table 3:** Mean substitution rate averaged across the whole tree for each analysis run in BEAST with 95% HPD and 25% burn-in.

Analysis	Mean rate	Lower and upper 95% HPD of mean rate
4pBD1d	1.96E-08	1.56E-08–2.38E-08
4pBD2d	2.04E-08	1.68E-08–2.40E-08
4pBDn1d	3.55E-08	1.76E-08–5.71E-08
4pY1d	1.17E-08	6.46E-09–1.69E-08
4pY2d	1.80E-08	1.43E-08–2.18E-08
4pYn1d	3.96E-08	1.94E-08–6.31E-08
5pBD1d	1.82E-08	1.48E-08–2.19E-08
5pBD2d	1.95E-08	1.63E-08–2.27E-08
5pY1d	1.52E-08	1.17E-08–1.87E-08
5pY2d	1.77E-08	1.45E-08–2.10E-08
5pYn1d	3.57E-08	1.92E-08–5.47E-08

Given in number of substitutions per site per million years 4p = 4 partitions; 5p = 5 partitions; BD = Births and deaths model; Y = Yule model; 2d = 2 calibration dates (Perissodactyla and Plains zebra); 1d = 1 calibration date (Perissodactyla); n = no outgroup (1d = Plains zebra).

Assuming only the more recent calibration, we find that caballine and non-caballine equids most likely diverged ∼4 Mya. This estimate is older than the age of the oldest fossil unambiguously identified as *Equus* in the paleontological record, which has been dated to 2.1 Mya [74]. According to our estimates, the mitochondrial TMRCA of the lineage leading to domestic horses (*E. caballus*) is ∼ 364 Kya (95% CI: 336–389 Kya). This is older than that reported in Steiner and Ryder [12] (250 Kya), Achilli et al. [5] (150 Kya), and Lippold et al. [7] (100 Kya), all of whom assumed that the genus *Equus* emerged ∼2 Mya. We should caution that our estimates assume 700 Ky as a calibration date for the TMRCA of Plains zebras. In the current dataset, the latter is estimated based on only three lineages, which likely leads to an under-estimation of the true phylogenetic distance among Plains zebras, and consequently of the mutation rate. Therefore, our molecular clock analyses are expected to provide upper bound estimates for divergence times and TMRCA dates. Additional complete mitochondrial genome data from modern Plain zebras coupled with tip calibration based on a series of radiocarbon dated ancient equid samples [75] will be needed to revisit the exact timing of lineage radiation in equids. For now, we note that most of the extant equid lineages radiate at the transition from the Tertiary to the Quaternary at 2.6 Mya, with a TMRCA for caballine horses estimated at 2.6 Mya (95% CI: 2.4–2.9 Mya) (Table 2; Table S9b). The mitochondrial TMRCA for zebras dates back to 1.75 Mya (95% CI: 0.7–2.5 Mya) with the Grevy’s and Plains zebras diverging 1.5 Mya; this is similar to estimates by Steiner and Ryder [12] of 1.2 Mya and George and Ryder [60] of 1.6 Mya. We estimate that African asses shared a TMRCA 713 Kya (95% CI: 706–723 Kya), in agreement with the date Krüger et al. [3] estimated using nuclear data (0.5–1.5 Mya). Finally, within Asiatic wild asses, we estimate that the mitochondrial divergence between the Kiang and Onager/Kulan lineages occurred 672 Kya (95% CI: 649–688 Kya). This time depth supports a separate species status for the Kiang, previously proposed on the basis of morphological [76] and chromosomal distinctions (2n = 51–52 in *E. kiang* versus 2n = 54–56 in *E. hemionus*) [77].

### Concluding Remarks

Here, we provide new data to assess the timing of the appearance of extant and several Late Pleistocene equid lineages, and to investigate their evolutionary relationships to one another based on complete mitogenome sequences. Our results are relevant to taxonomic boundaries: (1) While we confirm the monophyly of zebras, we find a surprisingly deep evolutionary divergence between the Mountain zebra and Plains zebra; (2) our results support recognition of the Kiang as an evolutionarily distinct species, rather than part of a single radiation that includes the Onager and Kulan; (3) we confirm that New World stilt-legged horses are closely related to caballine horses; (4) we show that the enigmatic Late Pleistocene Sussemione is a non-caballine equid that is only distantly related to extant equid lineages; and (5) we confirm that the recently extinct Quagga of South Africa was a subspecies of the widespread Plains zebra. Our study has also revealed a rapid radiation within non-caballine equids, within which details regarding order and pattern of diversification could not be resolved from complete mitogenomes. The massive throughput of current NGS platforms now enables the characterization of the complete nuclear genome of non-model organisms [78]. Such genome-wide approaches will likely provide enough information to resolve the nodes that are presently still problematic.

## Supporting Information

Figure S1 **Constrained topology used for positive selection tests in PAML.** Branch numbers are shown and are equivalent to branch numbers used in Table S7.
(TIFF)

Figure S2 **The 12 topologies tested in the topological test.** Topology number 1 to 12 is equivalent to item number in Table S2. Suss = Sussemione (*E. ovodovi*); Burch = Plains zebra (*E. quagga*); Grev = Grevy’s zebra (*E. grevyi*); Moun = Mountain zebra (*E. zebra*); Ass = African wild ass and domestic donkey (*E. africanus* and *E. asinus);* Ona/Kul = *E. hemionus* (Onager and Kulan).
(PDF)

Figure S3 **DNA fragmentation and Nucleotide misincorporation patterns.** Panel A: Ancient sample ACAD2304 *E. ovodovi*. Panel B: Ancient sample QH1 *E. quagga quagga* (only shotgun reads are considered in order to avoid the base composition bias resulting from the target-enrichment approach). Panel C: MS272 *E. sp. NWSLH* Panel D: Modern sample H11 *E. zebra hartmannae*. The base composition of the reads is reported for the first 10 nucleotides sequenced (left: 1–10) as well as for the 10 nucleotides located upstream of the genomic region aligned to the reads (left: −1 to −10). In addition, the base composition of the last 10 nucleotides sequenced (right: −10 to −1) and of the 10 nucleotides located downstream from the reads (right: 1–10) is also provided, all in relation to the mitochondrial horse reference genome. Nucleotide positions located within reads are reported with a gray frame. Each dot reports the average base composition per position. The figure shows two parallel base compositions; these correspond to the base composition of the two mitochondrial DNA strands, one being relatively enriched in purines (H, heavy), compared to the other one (L, light). The frequencies of all possible mismatches and indels observed between the horse genome and the reads are reported in gray as a function of distance from 5′- to 3′-ends (first 25 nucleotides sequenced) and 3′- to 5′- (last 25 nucleotides), except for C>T and G>A that are reported in red and blue, respectively. These frequencies are calculated by dividing the total number of occurrences of the modified base at a given position in a read by the total number of the unmodified base at the same position in the horse genome.
(PDF)

Table S1 **Published mitogenomes from Genbank used in analyses.** Includes both equids and non-equid outgroups.
(PDF)

Table S2 **Topological test results.** Table displaying results of the 12 different topologies tested. The topologies are visually illustrated in Figure S2. Item = Topology number (Figure S2).
(PDF)

Table S3 **Selected models from jModelTest.** The best model selected by jModelTest per gene or codon is displayed according to analysis. A: On full dataset. B: On dataset excluding non-equid outgroup.
(PDF)

Table S4 **Primer pairs used to amplify some of the modern mitogenomes.** Ta = annealing temperature, Ext. = extension time, Size = size of the amplicon generated by the primer pair, Location = a rough estimate of the part of the mitogenome the primers amplify based on the horse reference mitogenome (JN398398). * SF and LF are universal mammalian mitogenome amplifying primers (designed in-house by Sandra Abel Nielsen), Pr1 and Pr2 are from study Xu et al. [17], and Pr3 was designed in-house by Ludovic Orlando.
(PDF)

Table S5 **Primer sets used to amplify 5 modern mitogenomes for FLX sequencing.** Primer set names as from Table S4, primer sets from Pr3 to 14.3_Pr3 make up shorter regions of the LF fragment. Pr1 and Pr2 are shorter primers to cover the hypervariable region of the control region, while the last four are to fill in gaps throughout the mitogenome.
(PDF)

Table S6 **Nucleotide frequency composition of all samples.** Nucleotide frequencies in percent of total number of nucleotides (excluding tandem repeats).
(PDF)

Table S7 **Results from branch selection tests.** dN/dS for each branch given per gene, calculated from PAML under Model 2 (with Model 0 values in the second to last bottom row). Branch numbers are as in Figure S1. Values superior to 1 are highlighted in bold. P-values from LRT are given in the bottom row, with p-values ≤0.02 highlighted in bold.
(PDF)

Table S8 **Pair-wise Distance results.** Uncorrected pairwise distances observed between Sussemione (*E. ovodovi*) and other equid species.
(PDF)

Table S9 **Mean node ages estimated from BEAST.** Total number of states is measured in millions of states (M). Node letter in parentheses corresponds to Figure 1. 4p = 4 partitions; 5p = 5 partitions; BD = Births and Deaths; Y = Yule model; Sum. = Sumatran rhino; Woolly = woolly rhino; NWSLH = New World stilt-legged horse; Asses = *E. hemionus* and *E. kiang*; Zeb = zebras; Donkey = *E. africanus* and *E. asinus,* Don = Donkey. A: Results are with a 25% burn-in for eight different datasets including outgroup. 1d = 1 date (Perissodactyla); 2d = 2 dates (Perissodactyla and Plains zebra). B: Results are with a 25% burn-in for 3 different datasets without outgroup. 1d = 1 date (Plains zebra); n = no outgroup.
(PDF)

## References

[1] Macfadden BJ (1986) Fossil horses from “Eohippus” (Hyracotherium) to Equus*:* scaling, Cope’s Law, and the evolution of body size. Paleobiology 12: 355–369.

[2] MacFadden BJ (2005) Fossil horses - Evidence for evolution. Science 307: 1728–1730. 15774746 10.1126/science.1105458

[3] Kruger K, Gaillard C, Stranzinger G, Rieder S (2005) Phylogenetic analysis and species allocation of individual equids using microsatellite data. Journal of Animal Breeding and Genetics 122: 78–86. 16130461 10.1111/j.1439-0388.2005.00505.x

[4] Groves C, Grubb P (2011) Ungulate taxonomy: Johns Hopkins University Press.

[5] Achilli A, Olivieri A, Soares P, Lancioni H, Hooshiar Kashani B, et al (2012) Mitochondrial genomes from modern horses reveal the major haplogroups that underwent domestication. Proc Natl Acad Sci U S A 109: 2449–2454. 22308342 10.1073/pnas.1111637109 PMC3289334

[6] Goto H, Ryder OA, Fisher AR, Schultz B, Pond SLK, et al (2011) A Massively Parallel Sequencing Approach Uncovers Ancient Origins and High Genetic Variability of Endangered Przewalski’s Horses. Genome Biology and Evolution 3: 1096–1106. 21803766 10.1093/gbe/evr067 PMC3194890

[7] Lippold S, Matzke NJ, Reissmann M, Hofreiter M (2011) Whole mitochondrial genome sequencing of domestic horses reveals incorporation of extensive wild horse diversity during domestication. Bmc Evolutionary Biology 11. 10.1186/1471-2148-11-328 PMC3247663 22082251

[8] McCue ME, Bannasch DL, Petersen JL, Gurr J, Bailey E, et al.. (2012) A High Density SNP Array for the Domestic Horse and Extant Perissodactyla: Utility for Association Mapping, Genetic Diversity, and Phylogeny Studies. Plos Genetics 8. 10.1371/journal.pgen.1002451 PMC3257288 22253606

[9] Oakenfull EA, Clegg JB (1998) Phylogenetic relationships within the genus Equus and the evolution of alpha and theta globin genes. Journal of Molecular Evolution 47: 772–783. 9847419 10.1007/pl00006436

[10] Oakenfull EA, Lim HN, Ryder OA (2000) A survey of equid mitochondrial DNA: Implications for the evolution, genetic diversity and conservation of Equus. Conservation Genetics 1: 341–355.

[11] Oakenfull EA, Ryder OA (1998) Mitochondrial control region and 12S rRNA variation in Przewalski’s horse (Equus przewalskii). Animal Genetics 29: 456–459. 9883508 10.1046/j.1365-2052.1998.296380.x

[12] Steiner CC, Ryder OA (2011) Molecular phylogeny and evolution of the Perissodactyla. Zoological Journal of the Linnean Society 163: 1289–1303.

[13] Wallner B, Brem G, Muller M, Achmann R (2003) Fixed nucleotide differences on the Y chromosome indicate clear divergence between Equus przewalskii and Equus caballus. Animal Genetics 34: 453–456. 14687077 10.1046/j.0268-9146.2003.01044.x

[14] Warmuth V, Eriksson A, Bower MA, Barker G, Barrett E, et al (2012) Reconstructing the origin and spread of horse domestication in the Eurasian steppe. Proceedings of the National Academy of Sciences of the United States of America 109: 8202–8206. 22566639 10.1073/pnas.1111122109 PMC3361400

[15] Jiang QY, Wei YM, Huang YN, Jiang HS, Guo YF, et al (2011) The complete mitochondrial genome and phylogenetic analysis of the Debao pony (Equus caballus). Molecular Biology Reports 38: 593–599. 20390359 10.1007/s11033-010-0145-8

[16] Xu S, Luosang J, Hua S, He J, Ciren A, et al (2007) High altitude adaptation and phylogenetic analysis of Tibetan horse based on the mitochondrial genome. Journal of Genetics and Genomics 34: 720–729. 17707216 10.1016/S1673-8527(07)60081-2

[17] Xu XF, Arnason U (1994) The Complete Mitochondrial-DNA Sequence of the Horse, Equus-Caballus - Extensive Heteroplasmy of the Control Region. Gene 148: 357–362. 7958969 10.1016/0378-1119(94)90713-7

[18] Luo Y, Chen Y, Liu FY, Jiang CH, Gao YQ (2011) Mitochondrial genome sequence of the Tibetan wild ass (Equus kiang). Mitochondrial DNA 22: 6–8. 21732718 10.3109/19401736.2011.588221

[19] Xu XF, Gullberg A, Arnason U (1996) The complete mitochondrial DNA (mtDNA) of the donkey and mtDNA comparisons among four closely related mammalian species-pairs. Journal of Molecular Evolution 43: 438–446. 8875857 10.1007/BF02337515

[20] Weinstock J, Willerslev E, Sher A, Tong WF, Ho SYW, et al (2005) Evolution, systematics, and phylogeography of Pleistocene horses in the New World: A molecular perspective. Plos Biology 3: 1373–1379. 10.1371/journal.pbio.0030241 PMC1159165 15974804

[21] Eisenmann V, Sergej V (2011) Unexpected finding of a new Equus species (Mammalia Perissodactyla) belonging to a supposedly extinct subgenus in late Pleistocene deposits of Khakassia (southwestern Siberia). Geodiversitas 33: 519–530.

[22] Orlando L, Metcalf JL, Alberdi MT, Telles-Antunes M, Bonjean D, et al (2009) Revising the recent evolutionary history of equids using ancient DNA. Proceedings of the National Academy of Sciences of the United States of America 106: 21754–21759. 20007379 10.1073/pnas.0903672106 PMC2799835

[23] Higuchi R, Bowman B, Freiberger M, Ryder OA, Wilson AC (1984) DNA-Sequences from the Quagga, an Extinct Member of the Horse Family. Nature 312: 282–284. 6504142 10.1038/312282a0

[24] Leonard JA, Rohland N, Glaberman S, Fleischer RC, Caccone A, et al (2005) A rapid loss of stripes: the evolutionary history of the extinct quagga. Biology Letters 1: 291–295. 17148190 10.1098/rsbl.2005.0323 PMC1617154

[25] Geigl EM, Grange T (2012) Eurasian wild asses in time and space: Morphological versus genetic diversity. Annals of Anatomy-Anatomischer Anzeiger 194: 88–102. 10.1016/j.aanat.2011.06.002 21820882

[26] Orlando L, Eisenmann V, Reynier F, Sondaar P, Hanni C (2003) Morphological convergence in Hippidion and Equus (Amerhippus) South American equids elucidated by ancient DNA analysis. Journal of Molecular Evolution 57: S29–S40. 15008401 10.1007/s00239-003-0005-4

[27] Orlando L, Mashkour M, Burke A, Douady CJ, Eisenmann V, et al (2006) Geographic distribution of an extinct equid (Equus hydruntinus : Mammalia, Equidae) revealed by morphological and genetical analyses of fossils. Molecular Ecology 15: 2083–2093. 16780426 10.1111/j.1365-294X.2006.02922.x

[28] Lorenzen ED, Arctander P, Siegismund HR (2008) High variation and very low differentiation in wide ranging plains zebra (Equus quagga): Insights from mtDNA and microsatellites. Molecular ecology 17: 2812–2824. 18466230 10.1111/j.1365-294X.2008.03781.x

[29] Vilstrup JT, Ho SYW, Foote AD, Morin PA, Kreb D, et al (2011) Mitogenomic phylogenetic analyses of the Delphinidae with an emphasis on the Globicephalinae. BMC evolutionary biology 11: 65. 21392378 10.1186/1471-2148-11-65 PMC3065423

[30] Lindgreen S (2012) AdapterRemoval: Easy Cleaning of Next Generation Sequencing Reads. BMC Research Notes 5: 337. 22748135 10.1186/1756-0500-5-337 PMC3532080

[31] Li H, Durbin R (2009) Fast and accurate short read alignment with Burrows-Wheeler transform. Bioinformatics 25: 1754–1760. 19451168 10.1093/bioinformatics/btp324 PMC2705234

[32] Schubert M, Ginolhac A, Lindgreen S, Thompson JF, Al-Rasheid KAS, et al (2012) Improving ancient DNA read mapping against modern reference genomes. BMC genomics 13: 178. 22574660 10.1186/1471-2164-13-178 PMC3468387

[33] Gilbert MT, Tomsho LP, Rendulic S, Packard M, Drautz DI, et al (2007) Whole-genome shotgun sequencing of mitochondria from ancient hair shafts. Science 317: 1927–1930. 17901335 10.1126/science.1146971

[34] Kampmann ML, Fordyce SL, Avila-Arcos MC, Rasmussen M, Willerslev E, et al (2011) A simple method for the parallel deep sequencing of full influenza A genomes. Journal of Virological Methods 178: 243–248. 21946287 10.1016/j.jviromet.2011.09.001

[35] Meyer M, Kircher M (2010) Illumina sequencing library preparation for highly multiplexed target capture and sequencing. Cold Spring Harbor Protocols 2010: pdb. prot5448. 10.1101/pdb.prot5448 20516186

[36] Maricic T, Whitten M, Pääbo S (2010) Multiplexed DNA sequence capture of mitochondrial genomes using PCR products. PLoS One 5: e14004. 21103372 10.1371/journal.pone.0014004 PMC2982832

[37] Ginolhac A, Rasmussen M, Gilbert MTP, Willerslev E, Orlando L (2011) mapDamage: testing for damage patterns in ancient DNA sequences. Bioinformatics 27: 2153–2155. 21659319 10.1093/bioinformatics/btr347

[38] Rohland N, Siedel H, Hofreiter M (2010) A rapid column-based ancient DNA extraction method for increased sample throughput. Molecular ecology resources 10: 677–683. 21565072 10.1111/j.1755-0998.2009.02824.x

[39] Kircher M (2012) Analysis of high-throughput ancient DNA sequencing data. Methods in molecular biology (Clifton, NJ) 840: 197–228. 10.1007/978-1-61779-516-9_23 22237537

[40] Li H, Handsaker B, Wysoker A, Fennell T, Ruan J, et al (2009) The Sequence Alignment/Map format and SAMtools. Bioinformatics 25: 2078–2079. 19505943 10.1093/bioinformatics/btp352 PMC2723002

[41] Stothard P (2000) The sequence manipulation suite: JavaScript programs for analyzing and formatting protein and DNA sequences. Biotechniques 28. 10.2144/00286ir01 10868275

[42] Guindon S, Gascuel O (2003) A simple, fast, and accurate algorithm to estimate large phylogenies by maximum likelihood. Systematic biology 52: 696–704. 14530136 10.1080/10635150390235520

[43] Posada D (2008) jModelTest: phylogenetic model averaging. Molecular biology and evolution 25: 1253–1256. 18397919 10.1093/molbev/msn083

[44] Tamura K, Peterson D, Peterson N, Stecher G, Nei M, et al (2011) MEGA5: molecular evolutionary genetics analysis using maximum likelihood, evolutionary distance, and maximum parsimony methods. Molecular biology and evolution 28: 2731–2739. 21546353 10.1093/molbev/msr121 PMC3203626

[45] Guindon S, Dufayard JF, Lefort V, Anisimova M, Hordijk W, et al (2010) New algorithms and methods to estimate maximum-likelihood phylogenies: assessing the performance of PhyML 3.0. Systematic biology 59: 307–321. 20525638 10.1093/sysbio/syq010

[46] Guindon S, Lethiec F, Duroux P, Gascuel O (2005) PHYML Online–a web server for fast maximum likelihood-based phylogenetic inference. Nucleic acids research 33: W557–W559. 15980534 10.1093/nar/gki352 PMC1160113

[47] Anisimova M, Gascuel O (2006) Approximate likelihood-ratio test for branches: A fast, accurate, and powerful alternative. Systematic biology 55: 539–552. 16785212 10.1080/10635150600755453

[48] Shimodaira H, Hasegawa M (2001) CONSEL: for assessing the confidence of phylogenetic tree selection. Bioinformatics 17: 1246–1247. 11751242 10.1093/bioinformatics/17.12.1246

[49] Silvestro D, Michalak I (2010) raxmlGUI: a graphical front-end for RAxML. Organisms Diversity & Evolution: 1–3.

[50] Ronquist F, Huelsenbeck JP (2003) MrBayes 3: Bayesian phylogenetic inference under mixed models. Bioinformatics 19: 1572–1574. 12912839 10.1093/bioinformatics/btg180

[51] Drummond AJ, Rambaut A (2007) BEAST: Bayesian evolutionary analysis by sampling trees. BMC evolutionary biology 7: 214. 17996036 10.1186/1471-2148-7-214 PMC2247476

[52] Rambaut A, Drummond A (2009) Tracer v1. 5: an MCMC trace analysis tool. Available: http://beast.bio.ed.ac.uk/. Accessed 1 December 2009.

[53] Prothero DR, Schoch RM (1989) The evolution of perissodactyls: Clarendon Press New York.

[54] Eisenmann V (1979) Evolutionary characters and phylogeny of the genus Equus (Mammalia, Perissodactyla). Comptes Rendus Hebdomadaires Des Seances De L Academie Des Sciences Serie D 288: 497–500.

[55] Eisenmann V (1980) Les chevaux (Equus sensu lato) fossiles et actuels: crânes et dents jugales supérieures: Éditions du Centre national de la recherche scientifique.

[56] Yang Z (2007) PAML 4: phylogenetic analysis by maximum likelihood. Molecular biology and evolution 24: 1586–1591. 17483113 10.1093/molbev/msm088

[57] Steiner CC, Mitelberg A, Tursi R, Ryder OA (2012) Molecular phylogeny of extant equids and effects of ancestral polymorphism in resolving species-level phylogenies. Molecular Phylogenetics and Evolution. 10.1016/j.ympev.2012.07.010 22846684

[58] Hailer F, Kutschera VE, Hallström BM, Klassert D, Fain SR, et al (2012) Nuclear genomic sequences reveal that polar bears are an old and distinct bear lineage. Science 336: 344–347. 22517859 10.1126/science.1216424

[59] Reich D, Green RE, Kircher M, Krause J, Patterson N, et al (2010) Genetic history of an archaic hominin group from Denisova Cave in Siberia. Nature 468: 1053–1060. 21179161 10.1038/nature09710 PMC4306417

[60] George M, Ryder OA (1986) Mitochondrial DNA evolution in the genus Equus. Molecular Biology and Evolution 3: 535–546. 2832696 10.1093/oxfordjournals.molbev.a040414

[61] Kaminski M (1979) The biochemical evolution of the horse. Comparative biochemistry and physiology B, Comparative biochemistry 63: 175. 400961 10.1016/0305-0491(79)90025-7

[62] Eisenmann V (2010) Sussemionus, a new subgenus of Equus (Perissodactyla, Mammalia). Comptes Rendus Biologies 333: 235–240. 20338542 10.1016/j.crvi.2009.12.013

[63] Hoelzer GA, Meinick DJ (1994) Patterns of speciation and limits to phylogenetic resolution. Trends in ecology & evolution 9: 104–107. 21236789 10.1016/0169-5347(94)90207-0

[64] Walsh H, Kidd M, Moum T, Friesen V (1999) Polytomies and the power of phylogenetic inference. Evolution: 932–937. 10.1111/j.1558-5646.1999.tb05386.x 28565639

[65] Maddison WP (1997) Gene trees in species trees. Systematic biology 46: 523–536.

[66] Bennett DK (1980) Stripes do not a zebra make, Part I: A cladistic analysis of Equus. Systematic Biology 29: 272–287.

[67] Eisenmann V (1979) Les chevaux (Equus sensu lato) fossiles et actuels: étude craniologique et odontologique. Th∼ se re doctorat d∼ état Univ Pierre et Marie Curie, Paris VI: 1–444.

[68] Lowenstein J, Ryder O (1985) Immunological systematics of the extinct quagga (Equidae). Cellular and Molecular Life Sciences 41: 1192–1193. 10.1007/BF01951724 4043335

[69] Price SA, Bininda-Emonds ORP (2009) A comprehensive phylogeny of extant horses, rhinos and tapirs (Perissodactyla) through data combination. Zoosystematics and Evolution 85: 277–292.

[70] Ho SYW, Larson G (2006) Molecular clocks: when timesare a-changin’. TRENDS in Genetics 22: 79–83. 16356585 10.1016/j.tig.2005.11.006

[71] Ho SYW, Phillips MJ, Cooper A, Drummond AJ (2005) Time dependency of molecular rate estimates and systematic overestimation of recent divergence times. Molecular biology and evolution 22: 1561–1568. 15814826 10.1093/molbev/msi145

[72] Ho SYW, Lanfear R, Bromham L, Phillips MJ, Soubrier J, et al (2011) Time-dependent rates of molecular evolution. Molecular ecology 20: 3087–3101. 21740474 10.1111/j.1365-294X.2011.05178.x

[73] Soubrier J, Steel M, Lee MSY, Sarkissian CD, Guindon S, et al.. (2012) The influence of rate heterogeneity among sites on the time dependence of molecular rates. Molecular Biology and Evolution. 10.1093/molbev/mss140 22617951

[74] Bell CJ, Lundelius EJ, Barnosky AD, Graham RW, Lindsay EH, et al.. (2004) The Blancan, Irvingtonian, and Rancholabrean mammal ages. In: Late Cretaceous and Cenozoic mammals of North America: biostratigraphy and geochronology; Woodburne MO, editor: Columbia University Press.

[75] Ho SYW, Saarma U, Barnett R, Haile J, Shapiro B (2008) The effect of inappropriate calibration: three case studies in molecular ecology. PLoS One 3: e1615. 18286172 10.1371/journal.pone.0001615 PMC2229648

[76] Groves C, Mazák V (1967) On some taxonomic problems of Asiatic wild asses; with the description of a new subspecies (Perissodactyla; Equidae). Zeitschrift für Säugetierkunde 32: 321–355.

[77] Musilova P, Kubickova S, Horin P, Vodička R, Rubes J (2009) Karyotypic relationships in Asiatic asses (kulan and kiang) as defined using horse chromosome arm-specific and region-specific probes. Chromosome Research 17: 783–790. 19731053 10.1007/s10577-009-9069-3

[78] Ekblom R, Galindo J (2010) Applications of next generation sequencing in molecular ecology of non-model organisms. Heredity 107: 1–15. 21139633 10.1038/hdy.2010.152 PMC3186121

