# Supplementary figures and images for: Mitochondrial Phylogenomics of Modern and Ancient Equids
Source: PLoS One. 2013 Feb 20;8(2):e55950. doi: 10.1371/journal.pone.0055950 (PMC3577844; doi:10.1371/journal.pone.0055950)

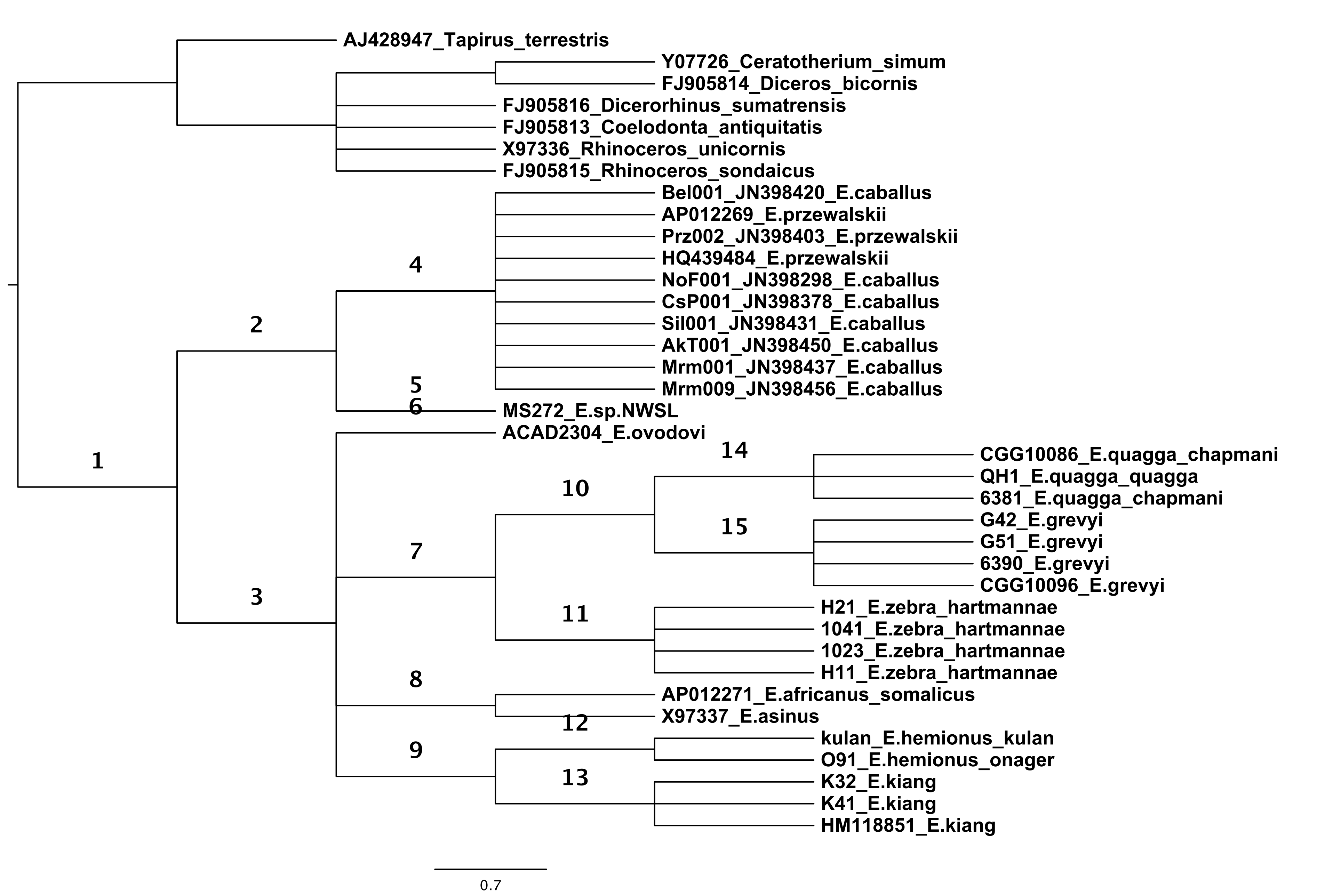

Supplement: Figure S1 — Constrained topology used for positive selection tests in PAML. Branch numbers are shown and are equivalent to branch numbers used in Table S7. (TIFF) [file pone.0055950.s001.tif]

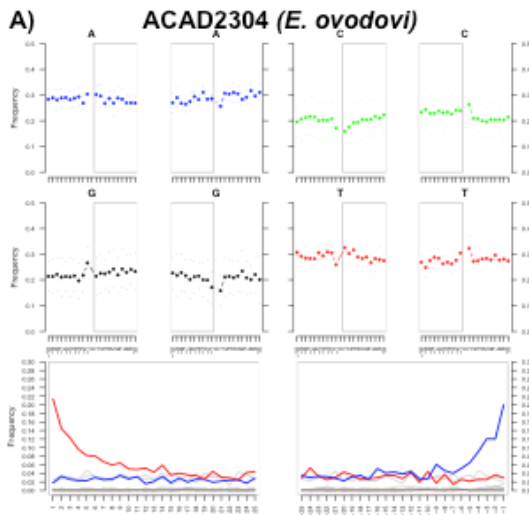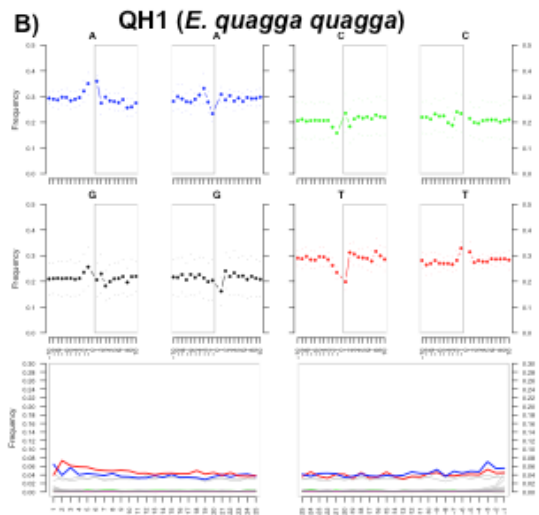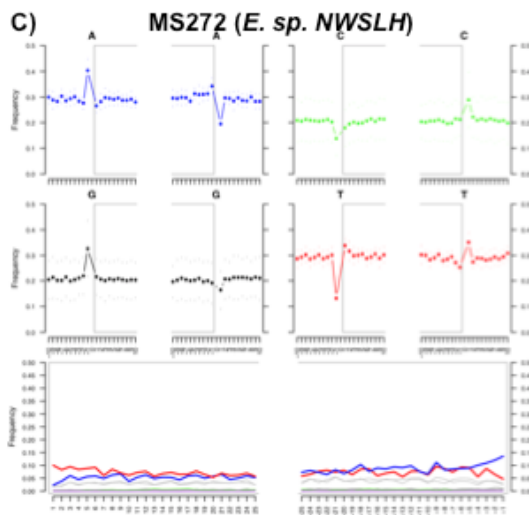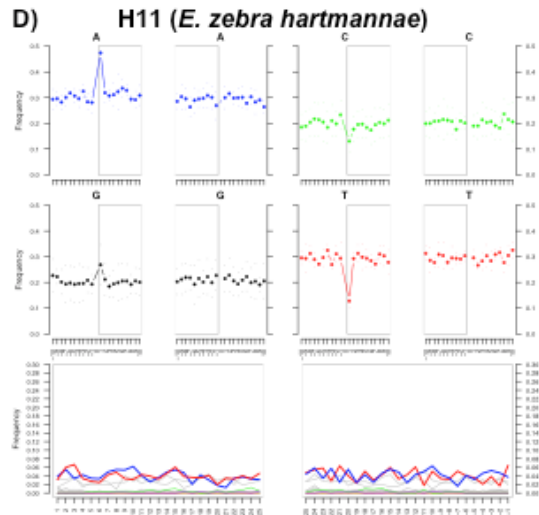

Supplement: Figure S3 — DNA fragmentation and Nucleotide misincorporation patterns. Panel A: Ancient sample ACAD2304 E. ovodovi. Panel B: Ancient sample QH1 E. quagga quagga (only shotgun reads are considered in order to avoid the base composition bias resulting from the target-enrichment approach). Panel C: MS272 E. sp. NWSLH Panel D: Modern sample H11 E. zebra hartmannae. The base composition of the reads is reported for the first 10 nucleotides sequenced (left: 1–10) as well as for the 10 nucleotides located upstream of the genomic region aligned to the reads (left: −1 to −10). In addition, the base composition of the last 10 nucleotides sequenced (right: −10 to −1) and of the 10 nucleotides located downstream from the reads (right: 1–10) is also provided, all in relation to the mitochondrial horse reference genome. Nucleotide positions located within reads are reported with a gray frame. Each dot reports the average base composition per position. The figure shows two parallel base compositions; these correspond to the base composition of the two mitochondrial DNA strands, one being relatively enriched in purines (H, heavy), compared to the other one (L, light). The frequencies of all possible mismatches and indels observed between the horse genome and the reads are reported in gray as a function of distance from 5′- to 3′-ends (first 25 nucleotides sequenced) and 3′- to 5′- (last 25 nucleotides), except for C>T and G>A that are reported in red and blue, respectively. These frequencies are calculated by dividing the total number of occurrences of the modified base at a given position in a read by the total number of the unmodified base at the same position in the horse genome. (PDF) [file pone.0055950.s003.pdf]
