# Supplementary material for: Mitochondrial Phylogenomics of Modern and Ancient Equids
Source: PLoS One. 2013 Feb 20;8(2):e55950. doi: 10.1371/journal.pone.0055950 (PMC3577844; doi:10.1371/journal.pone.0055950)
Supplement: Figure S2 — The 12 topologies tested in the topological test. Topology number 1 to 12 is equivalent to item number in Table S2. Suss = Sussemione (E. ovodovi); Burch = Plains zebra (E. quagga); Grev = Grevy’s zebra (E. grevyi); Moun = Mountain zebra (E. zebra); Ass = African wild ass and domestic donkey (E. africanus and E. asinus); Ona/Kul = E. hemionus (Onager and Kulan). (PDF) [file pone.0055950.s002.pdf]

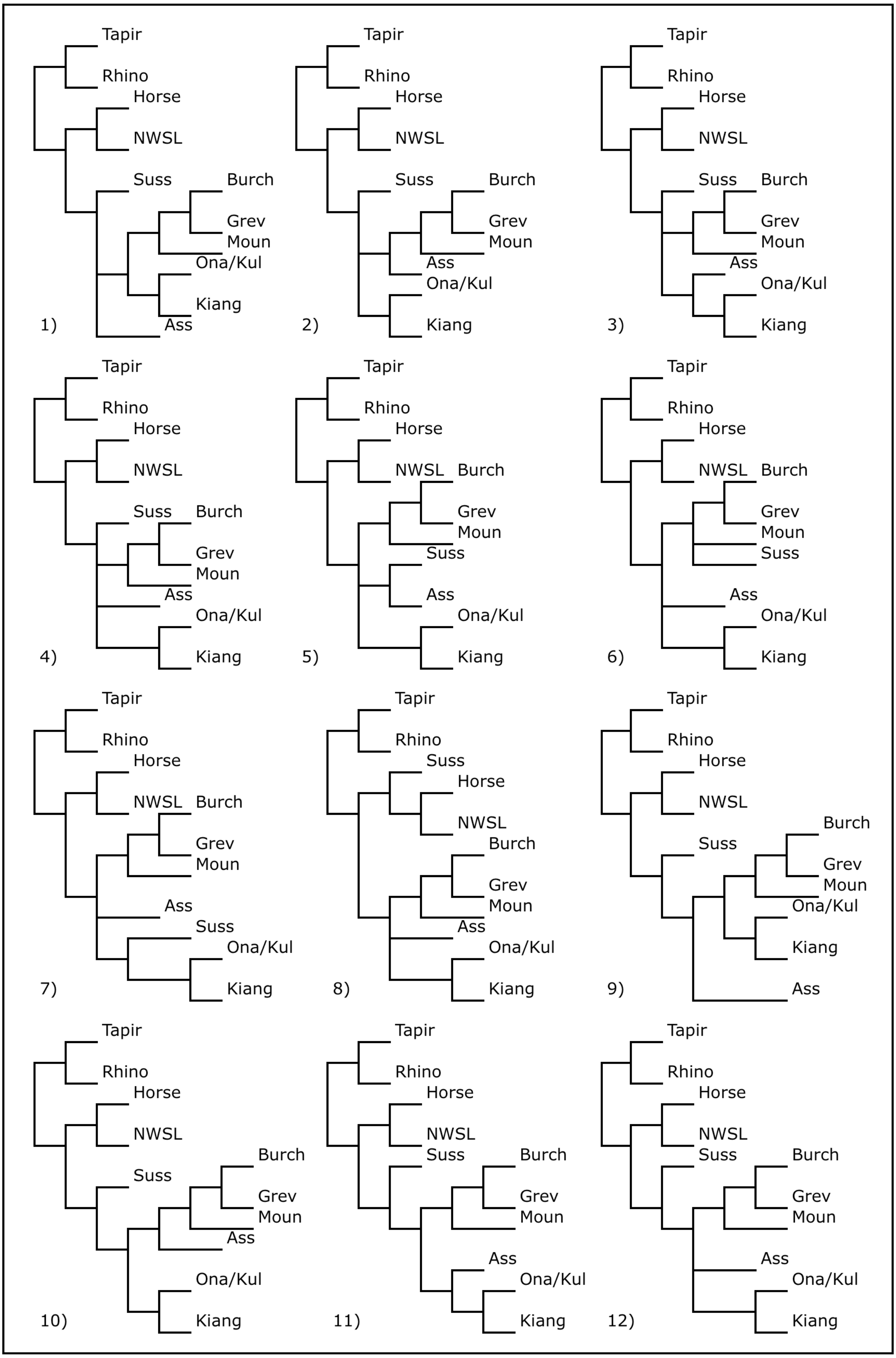

Figure S2: The 12 topologies tested in the topological test. Topology number 1 to 12 is equivalent to item number in Table S2. Suss = Sussemione (*E. ovodovi*); Burch = Plains zebra (*E. quagga*); Grev = Grevy's zebra (*E. grevyi*); Moun = Mountain zebra (*E. zebra*); Ass = African wild ass and domestic donkey (*E. africanus* and *E. asinus*); Ona/Kul = *E. hemionus* (Onager and Kulan).
