# Supplementary material for: Mitochondrial Phylogenomics of Modern and Ancient Equids
Source: PLoS One. 2013 Feb 20;8(2):e55950. doi: 10.1371/journal.pone.0055950 (PMC3577844; doi:10.1371/journal.pone.0055950)
Supplement: Table S1 — Published mitogenomes from Genbank used in analyses. Includes both equids and non-equid outgroups. (PDF) [file pone.0055950.s004.pdf]

Table S1: Published mitogenomes used in analyses from Genbank. Includes both equids and non-equid outgroups.

Sample name	Species name	Common name/breed	Location	Genbank acc. no.	Reference
Bel001	*Equus caballus*	Belgian draft	Europe centre	JN398420	[19]
NoF001	*E. caballus*	Norwegian fjord	Europe North	JN398398	[19]
CsP001	*E. caballus*	Caspian pony	Middle East	JN398378	[19]
Sil001	*E. caballus*	Silensian	Europe centre	JN398431	[19]
Mrm009	*E. caballus*	Maremmano	Europe south	JN398456	[19]
AkT001	*E. caballus*	Akhal-Teke	Asia centre	JN398450	[19]
Mrm001	*E. caballus*	Maremmano	Europe south	JN398437	[19]
Prz002	*E. przewalskii*	Przewalski	Asia centre	JN398403	[19]
HQ439484	*E. przewalskii*	Przewalski	East Asia	HQ439484	[17]
AP012269	*E. przewalskii*	Przewalski	San Diego Zoo	AP012269	[14]
AP012271	*E. africanus somalicus*	Somalian wild ass	San Diego Zoo	AP012271	[14]
X97337	*E. asinus*	Domestic donkey	Glasgow Vet. Sc.	X97337	[25]
HM118851	*E. kiang*	Kiang	Tibet	HM118851	[24]
FJ905816	*Dicerorhinus sumatrensis*	Sumatran rhinoceros	Cincinatti Zoo	FJ905816	[90]
FJ905813	*Coelodonta antiquitatis*	Woolly rhinoceros	Yakutia, Russia	FJ905813	[90]
X97336	*Rhinoceros unicornis*	Indian rhinoceros	Berlin Zoo	X97336	[25]
FJ905815	*Rhinoceros sondaicus*	Javan rhninoceros	NHM, Uni. Oxford	FJ905815	[90]
Y07726	*Ceratotherium simum*	White rhinoceros	Uni. Copenhagen	Y07726	[91]
FJ905814	*Diceros bicornis*	Black rhinoceros	Copenhagen Zoo	FJ905814	[90]
AJ428947	*Tapirus terrestris*	Lowland tapir	Kolmården Zoo, Sweden	AJ428947	Janke et al. Unpubl.
