# Supplementary material for: Mitochondrial Phylogenomics of Modern and Ancient Equids
Source: PLoS One. 2013 Feb 20;8(2):e55950. doi: 10.1371/journal.pone.0055950 (PMC3577844; doi:10.1371/journal.pone.0055950)
Supplement: Table S2 — Topological test results. Table displaying results of the 12 different topologies tested. The topologies are visually illustrated in Figure S2. Item = Topology number (Figure S2). (PDF) [file pone.0055950.s005.pdf]

Table S2: Topological test results. Table displaying results of the 12 different topologies tested. The topologies are visually illustrated in Figure S2. Item = Topology number (Figure S2).

rank	item	obs	au	np	bp	pp	kh	sh	wkh	wsh
1	11	-2.000	0.772	0.443	0.444	0.699	0.629	0.977	0.629	0.958
2	2	2.000	0.579	0.232	0.232	0.095	0.371	0.775	0.371	0.837
3	3	2.200	0.231	0.076	0.074	0.080	0.187	0.733	0.187	0.538
4	12	3.400	0.264	0.046	0.047	0.022	0.215	0.713	0.215	0.715
5	10	3.400	0.264	0.046	0.047	0.022	0.215	0.713	0.215	0.715
6	6	3.500	0.323	0.058	0.026	0.020	0.265	0.635	0.265	0.810
7	4	3.500	0.321	0.057	0.023	0.020	0.265	0.634	0.265	0.804
8	1	3.900	0.307	0.113	0.112	0.015	0.244	0.607	0.244	0.618
9	5	4.000	0.169	0.020	0.018	0.013	0.247	0.594	0.195	0.634
10	9	4.200	0.165	0.021	0.019	0.010	0.152	0.620	0.152	0.556
11	7	5.300	0.070	0.003	0.003	0.003	0.150	0.519	0.141	0.545
12	8	24.400	0.001	4E-04	4E-04	2E-11	0.006	0.016	0.006	0.016
