# Supplementary material for: Mitochondrial Phylogenomics of Modern and Ancient Equids
Source: PLoS One. 2013 Feb 20;8(2):e55950. doi: 10.1371/journal.pone.0055950 (PMC3577844; doi:10.1371/journal.pone.0055950)
Supplement: Table S3 — Selected models from jModelTest. The best model selected by jModelTest per gene or codon is displayed according to analysis. A: On full dataset. B: On dataset excluding non-equid outgroup. (PDF) [file pone.0055950.s006.pdf]

Table S3a: jModelTest model selection on full data set. The best model selected by jModelTest per gene or codon is displayed according to analysis.

Gene/Codon	Best model*	Best model according to PhyML	MrBayes
ND1	TrN+I+G	TrN+I+G	GTR+I+G
ND2	TIM1+I+G	TrN+I+G	GTR+I+G
COX1	GTR+I+G	GTR+I+G	GTR+I+G
COX2	TVM+I+G	GTR+I+G	GTR+I+G
ATP8	TrN+G	TrN+G	HKY+G
ATP6	TrN+I+G	TrN+I+G	GTR+I+G
COX3	TVM+I+G	GTR+I+G	GTR+I+G
ND3	TPM1uf+I+G	HKY+I+G	HKY+I+G
ND4L	HKY+G	HKY+G	HKY+G
ND4	GTR+I+G	GTR+I+G	GTR+I+G
ND5	GTR+I+G	GTR+I+G	GTR+I+G
ND6	TPM1uf+G	HKY+G	HKY+G
CYTB	TrN+I+G	TrN+I+G	HKY+I+G
CR	HKY+G	HKY+G	HKY+G
12sRNA	GTR+I+G	GTR+I+G	GTR+I+G
16sRNA	GTR+G	GTR+G	GTR+G
Codon1	GTR+I+G	GTR+I+G	GTR+I+G
Codon2	GTR+I+G	GTR+I+G	GTR+I+G
Codon3	TIM1+I+G	GTR+I+G	GTR+I+G
RNAs	GTR+I+G	GTR+I+G	GTR+I+G
unpart	TVM+I+G	GTR+I+G	GTR+I+G

* for RaxML and MrBayes GTR+I+G was chosen for both the 4 and the 5 partitions datasets.

Table S3b: jModelTest model selection on data set excluding non-equid outgroups. The best model selected by jModelTest per gene or codon is displayed according to analysis.

Gene/Codon	Best model*	Best model according to PhyML	MrBayes
ND1	HKY+G	HKY+G	HKY+G
ND2	TPM1uf+I	HKY+I	HKY+I
COX1	TPM1uf+I	GTR+I	GTR+I
COX2	TVM+G	GTR+G	GTR+G
ATP8	TrN+G	TrN+G	HKY+G
ATP6	TrN+I	TrN+I	GTR+I
COX3	TPM1uf+G	HKY+G	HKY+G
ND3	TPM1uf+I	HKY+I	HKY+I
ND4L	HKY+G	HKY+G	HKY+G
ND4	TIM1+I	GTR+I	GTR+I
ND5	GTR+I+G	GTR+I+G	GTR+I+G
ND6	TPM1uf+G	TrN+G	GTR+G
CYTB	TrN+I	TrN+I	GTR+I
CR	TrN+G	TrN+G	HKY+G
12sRNA	TIM1+G	GTR+G	GTR+G
16sRNA	TIM+I+G	TrN+I+G	GTR+I+G
Codon1	GTR+I+G	GTR+I+G	GTR+I+G
Codon2	TIM1+I+G	GTR+I+G	GTR+I+G
Codon3	TIM1+I+G	GTR+I+G	GTR+I+G
RNAs	TIM+I+G	GTR+I+G	GTR+I+G
unpart	TVM+G	GTR+I+G	GTR+I+G

* for RaxML and MrBayes GTR+I+G was chosen for both the 4 and the 5 partitions datasets excluding outgroups.
