# Supplementary material for: Mitochondrial Phylogenomics of Modern and Ancient Equids
Source: PLoS One. 2013 Feb 20;8(2):e55950. doi: 10.1371/journal.pone.0055950 (PMC3577844; doi:10.1371/journal.pone.0055950)
Supplement: Table S4 — Primer pairs used to amplify some of the modern mitogenomes. Ta = annealing temperature, Ext. = extension time, Size = size of the amplicon generated by the primer pair, Location = a rough estimate of the part of the mitogenome the primers amplify based on the horse reference mitogenome (JN398398). * SF and LF are universal mammalian mitogenome amplifying primers (designed in-house by Sandra Abel Nielsen), Pr1 and Pr2 are from study Xu et al. [17], and Pr3 was designed in-house by Ludovic Orlando. (PDF) [file pone.0055950.s007.pdf]

Table S4: Primer pairs used to amplify some of the modern mitogenomes. Ta = annealing temerature, Ext. = extension time, Size = size of the amplicon generated by the primer pair, Location = a rough estimate of the part of the mitogenome the primers amplify based on the horse reference mitogenome (JN398398).* SF and LF are universal mammalian mitogenome amplifying primers (designed in-house by Sandra Abel Nielsen), Pr1 and Pr2 are from study Xu et al. [19], and Pr3 was designed in-house by Ludovic Orlando.

Primer pair	Primer sequence	Ta °C	Ext.	Size (bp)	Location bp
LF_Fwd*<br>LF_Rev*	5'-CCGCCTGTTTACCAAAAACATCACC-3'<br>5'-CGAATTTTGARTTTCCTGRACCGCC-3'	59	16min	15,100	1,980-580
SF_Fwd*<br>SF_Rev*	5'-GCTTAAAACTYAAAGGACYTGGCGG-3'<br>5'-GGCGGACAAATGGTTTTTGTAGTGG-3'	59	1min 30s	1,400	580-1,980
Pr3_Fwd*<br>Pr3_Rev*	5'-CTCCCAAAAGCCCATGTAGA-3'<br>5'-GYCTACACCTTGACCTAACG-3'	50	6min 30s	6,500	10,800-750
LF_Fwd<br>Pr3.2_Rev	see above<br>5'-TGCTCGTCATTTAGGCTGTG-3'	50	11min	10,900	1,900-12,800
6.3_Fwd<br>11.3_Rev	5'-CACATCAGCTACCATAATCATCGC-3'<br>5'-GGTTAGGCTGGCTAATAGTCATC-3'	50	5min	5,000	6,300-11,300
1.6_Fwd<br>7.8_Rev	5'-GCAGCCATCAATTAAGAAAGCG-3'<br>5'-CATGTTGATGTATCCAACTGTGGC-3'	53	6min 30s	6,200	1,600-7,800
10.5_Fwd<br>15.4_Rev	5'-CCGCCACAGAACTAATCTCC-3'<br>5'-GTTGATGGTGGAGCTAGAGC-3'	50	5min	5,000	10,500-15,400
14.3_Fwd<br>Pr3_Rev	5'-CCTAGGAATCTGCCTAATCC-3'<br>see above	48	3min	3,000	14,300-750
Pr1_Fwd*<br>Pr1_Rev*	5'-TAACATGAATCGGCGGACA-3'<br>5'-TTGCTGAAGATGGCGGTAT-3'	57	90s	2,000	15,190-700
Pr2_Fwd*<br>Pr2_Rev*	5'-ATTTCCATAGACAGGCATCC-3'<br>5'-TCACCTCTACCTACGAATCTTCT-3'	57	90s	1,500	16,600-1,440
MAC_16.2_Fwd<br>MAC_16.4_Rev	5'-ATTACTCCGCATCAGCAACC-3'<br>5'-GGGGGTTTGATTARGAAARTTACA-3'	53	30s	200	16,200-16,400
MAC_1.4_Fwd<br>MAC_1.6_Rev	5'-TGTCGCAAAATAGTGAGAAGATTT-3'<br>5'-TGGCTGCTTTTAAGCCAACT-3'	53	30s	200	1,400-1,600
MAC_16.5_Fwd<br>MAC_16.6_Rev	5'-ACCCCRAAAACAAGACYAAA-3'<br>5'-TGYAGGTTTGGAAAATCAGA-3'	53	30s	100	16,500-16,600
MAC_1.8_Fwd<br>MAC_2.0_Rev	5'-CAACCCAAAAACTAACCACCT-3'<br>5'-TTTGCACGGTTAGGATACCG-3'	53	30s	200	1,800-2,000
0.7rev<br>1.0fwd<br>1.4rev<br>1.8fwd	5'-GATTAAGTCGGATATATGGCGG-3'<br>5'-GTATACCGGAAGGTGTACTTGG-3'<br>5'-CTTGGTTGAGTAGATACAGCG-3'<br>5'-GTTAACCCAACACAGGCATG-3'	(These were designed for sequencing at Macrogen together with the SF.Fwd and SF.Rev primers, and were never amplified in our laboratory)			
