# Supplementary material for: Mitochondrial Phylogenomics of Modern and Ancient Equids
Source: PLoS One. 2013 Feb 20;8(2):e55950. doi: 10.1371/journal.pone.0055950 (PMC3577844; doi:10.1371/journal.pone.0055950)
Supplement: Table S5 — Primer sets used to amplify 5 modern mitogenomes for FLX sequencing. Primer set names as from Table S4, primer sets from Pr3 to 14.3_Pr3 make up shorter regions of the LF fragment. Pr1 and Pr2 are shorter primers to cover the hypervariable region of the control region, while the last four are to fill in gaps throughout the mitogenome. (PDF) [file pone.0055950.s008.pdf]

Table S5: Primer sets used to amplify 5 modern mitogenomes for FLX sequencing. Primer set names as from Table S4, primer sets from Pr3 to 14.3+Pr3 t make up shorter regions of the LF fragment. Pr1 and Pr2 are shorter primers to cover the hypervavariable region of the control region,while the last four are to fill in gaps throughout the mitogenome.

**Sample**	**Species**	SF	LF	Pr3	LF+ Pr3.2	6.3+ 11.3	1.6+ 7.8	10.5+ 15.4	14.3+ Pr3	Pr1	Pr2	16.2+ 16.4	16.5+ 16.6	1.8+ 2.0	1.4+ 1.6
Kulan	*E. hemionus kulan*	x				x	x	x	x	x	x	x			x
1023	*E. zebra*	x		x	x	x	x			x			x		
1041	*E. zebra*	x	x			x	x	x	x	x			x		
6390	*E. grevyi*	x	x							x		x	x	x	
6381	*E. quagga chapmani*	x	x							x				x	
