# Supplementary material for: Mitochondrial Phylogenomics of Modern and Ancient Equids
Source: PLoS One. 2013 Feb 20;8(2):e55950. doi: 10.1371/journal.pone.0055950 (PMC3577844; doi:10.1371/journal.pone.0055950)
Supplement: Table S6 — Nucleotide frequency composition of all samples. Nucleotide frequencies in percent of total number of nucleotides (excluding tandem repeats). (PDF) [file pone.0055950.s009.pdf]

Table S6: Nucleotide frequency composition of all samples. Nucleotide frequencies in percent of total number of nucleotides (excluding tandem repeats).

Sample	T (U)	C	A	G	Total
JW328_E.sp.NWSLH	25.3	29.4	30.3	14.9	7035
ACAD2304_E.ovodovi	25.4	29	32.5	13.2	16166
MS272_E.sp.NWSLH	26.3	28	32.6	13.1	16042
X97337_E.asinus	25.9	28.5	32.4	13.2	16412
AP012271_E.a.somalicus	26	27.8	32.8	13.4	14083
AP012269_E.przewalskii_Bonnette	25.8	28.5	32.5	13.2	16155
HM118851_E.kiang	25.8	28.6	32.5	13.1	16423
Kulan_E.hemionus_kulan	25.9	28.4	32.6	13.1	16380
O91_E.hemionus_onager	25.8	28.6	32.5	13.1	16378
K41_E.kiang	25.8	28.6	32.5	13.1	16379
K32_E.kiang	25.8	28.6	32.5	13	16380
1023_E.zebra_hartmannae	25.9	28.6	32.3	13.3	16402
1041_E.zebra_hartmannae	25.8	28.6	32.3	13.3	16404
H11_E.zebra_hartmannae	26	28.5	32.3	13.3	16389
H21_E.zebra_hartmannae	25.8	28.6	32.3	13.3	16408
6390_E.grevyi	25.9	28.6	32.5	13.1	16402
CGG10096_E.grevyi	25.6	29	32.1	13.3	14711
G51_E.grevyi	25.9	28.6	32.5	13.1	16400
G42_E.grevyi	25.9	28.5	32.5	13.1	16389
6381_E.quagga_chapmani	26	28.5	32.5	13.1	16406
QUAGGa_E.quagga_quagga	26	28.4	32.4	13.2	16364
CGG10086_E.quagga_chapmani	26	28.5	32.4	13.2	16243
HQ439484_E.przewalskii	25.9	28.4	32.5	13.2	16415
CsP001_E.caballus_A	25.9	28.4	32.5	13.2	16412
NoF001_E.caballus_D	25.9	28.4	32.5	13.2	16411
Prz002_E.caballus_F	25.9	28.4	32.5	13.2	16411
Bel001_E.caballus_K	25.9	28.5	32.5	13.2	16411
Sil001_E.caballus_L	25.9	28.4	32.4	13.3	16410
Mrm001_E.caballus_M	25.9	28.4	32.5	13.2	16411
Akt001_E.caballus_Q	25.8	28.5	32.4	13.3	16411
Mrm009_E.caballus_R	25.9	28.4	32.5	13.2	16411
FJ905815_R.sondaicus	26.3	27.3	33.6	12.7	16364
FJ905813_C.antiquitatis	26.4	27.3	33.5	12.8	16381
FJ905814_D.bicornis	26	27.9	33.4	12.7	16358
AJ428947_T.terrestris	28	25.2	34.2	12.6	16377
X97336_R.unicornis	26.3	27.4	33.6	12.6	16430
Y07726_C.simum	25.9	27.9	33.3	13	16398
FJ905816_D.sumatrensis	25.4	28.2	33.1	13.3	16419
