# Supplementary material for: Mitochondrial Phylogenomics of Modern and Ancient Equids
Source: PLoS One. 2013 Feb 20;8(2):e55950. doi: 10.1371/journal.pone.0055950 (PMC3577844; doi:10.1371/journal.pone.0055950)
Supplement: Table S7 — Results from branch selection tests. dN/dS for each branch given per gene, calculated from PAML under Model 2 (with Model 0 values in the second to last bottom row). Branch numbers are as in Figure S1. Values superior to 1 are highlighted in bold. P-values from LRT are given in the bottom row, with p-values ≤0.02 highlighted in bold. (PDF) [file pone.0055950.s010.pdf]

Table S7: Results from branch selection tests. dN/dS for each branch given per gene, calculated from PAML under Model 2 (with Model 0 values in the second to last bottom row). Branch numbers are as in Figure S1. Values superior to 1 are highlighted in bold. P-values from LRT are given in the bottom row, with p-values ≤0.02 highlighted in bold.

Branch	ATP6	ATP8	COX1	COX2	COX3	CYTB	ND1	ND2	ND3	ND4	ND4L	ND5	ND6
1	0.0050	0.0001	0.8566	0.0021	0.7984	0.3160	0.0067	0.8306	0.0102	0.0096	0.0001	0.0001	**2.4777**
2	0.0343	0.0001	0.0113	0.0001	0.0001	0.0001	0.0001	0.0001	0.0001	**635.67**	0.0001	0.0147	0.0001
3	**999.00**	0.2548	0.0001	0.0001	**4.8396**	0.0001	0.0001	0.0180	0.0001	0.0001	0.0001	0.9820	0.0001
4	0.0591	**1.1179**	0.0001	0.0001	0.0111	0.0181	0.0287	0.0327	0.0281	0.0725	0.0378	0.0809	0.0919
5	0.0139	0.0806	0.0040	0.0001	0.0001	0.0146	0.0001	0.0294	0.0445	0.0715	0.0617	0.0272	0.0289
6	0.0604	0.1083	0.0001	0.0001	0.0256	0.0001	0.0170	0.0333	0.0443	0.0365	0.0001	0.0001	0.0001
7	0.0001	0.0001	0.0001	0.0001	0.0001	0.0001	0.0461	0.2324	0.0001	0.0001	0.5323	0.0001	0.0001
8	0.0414	0.8696	0.0051	0.0169	0.0001	0.0119	0.0244	0.0667	0.0001	0.0604	0.1282	0.0436	0.1474
9	0.0318	0.4379	0.0001	0.0001	0.0001	0.0001	0.0001	0.0227	0.0001	0.0503	0.0001	0.0430	0.0898
10	0.0001	0.8466	0.0001	0.0001	0.0001	0.0001	0.0001	0.0001	0.0001	0.0611	0.1734	0.0001	0.0001
11	0.0552	0.0001	0.0001	0.0001	0.0373	0.0001	0.0001	0.1344	0.0265	0.0001	0.0001	0.2001	0.1346
12	0.0001	**999.00**	0.0001	0.0001	0.0344	0.0616	0.0001	0.0452	0.0001	0.1319	0.1109	0.0184	0.0650
13	0.0001	**999.00**	0.0001	0.2355	0.1389	0.0360	0.0234	0.1636	0.0001	0.1972	0.0001	0.0344	0.1044
14	0.0585	0.1469	0.0001	0.0001	0.0001	0.0209	0.0119	0.0466	0.0001	0.0001	0.0001	0.0390	0.1197
15	0.0469	**999.00**	0.0001	0.0001	0.0001	0.0116	0.0001	0.0905	0.0001	0.0408	0.0001	0.0001	0.0001
Model 0	0.0356	0.2802	0.0048	0.0098	0.0068	0.0103	0.0109	0.0386	0.0274	0.0353	0.0287	0.0529	0.0804
LRT p-values	0.15	>0.25	>0.25	**0.02**	>0.25	>0.25	0.15	**0.005**	>0.25	**0.02**	0.25	**0.005**	>0.25
