# Supplementary material for: Mitochondrial Phylogenomics of Modern and Ancient Equids
Source: PLoS One. 2013 Feb 20;8(2):e55950. doi: 10.1371/journal.pone.0055950 (PMC3577844; doi:10.1371/journal.pone.0055950)
Supplement: Table S8 — Pair-wise Distance results. Uncorrected pairwise distances observed between Sussemione (E. ovodovi) and other equid species. (PDF) [file pone.0055950.s011.pdf]

Table S8: Pair-wise Distance results.
Uncorrected pair-wise distances observed between Sussemione and other equid species.

X97337_E.a.asinus	0.046
AP012271_E.a.somalicus	0.043
HM118851_E.kiang	0.039
kulan_E.hemionus_kulan	0.042
091_E.hemionus_onager	0.039
K41_E.kiang	0.039
K32_E.kiang	0.040
1023_E.zebra	0.046
1041_E.zebra	0.046
H11_E.zebra_hartmanni	0.047
H21_E.zebra_hartmanni	0.046
6390_E.grevyi	0.042
CGG10096_E.grevyi	0.043
G51_E.grevyi	0.042
G42_E.grevyi	0.042
6381_E.quagga_burchellii	0.042
QH1_E.quagga_quagga	0.043
CGG10086_E.quagga_burchellii	0.043
HQ439484_E.przewalskii	0.053
CsP001_E.caballus_A	0.054
NoF001_E.caballus_D	0.053
Prz002_E.przewalskii_F	0.053
Bel001_E.caballus_K	0.054
Sil001_E.caballus_L	0.053
Mrm001_E.caballus_M	0.056
Akt001_E.caballus_Q	0.054
Mrm009_E.caballus_R	0.055
JW328_E.sp.NWSL	0.063
M272_E.sp.NWSL	0.063
AP012269_E.przewalskii	0.054
