# Supplementary material for: Mitochondrial Phylogenomics of Modern and Ancient Equids
Source: PLoS One. 2013 Feb 20;8(2):e55950. doi: 10.1371/journal.pone.0055950 (PMC3577844; doi:10.1371/journal.pone.0055950)
Supplement: Table S9 — Mean node ages estimated from BEAST. Total number of states is measured in millions of states (M). Node letter in parentheses corresponds to Figure 1. 4p = 4 partitions; 5p = 5 partitions; BD = Births and Deaths; Y = Yule model; Sum. = Sumatran rhino; Woolly = woolly rhino; NWSLH = New World stilt-legged horse; Asses = E. hemionus and E. kiang; Zeb = zebras; Donkey = E. africanus and E. asinus, Don = Donkey. A: Results are with a 25% burn-in for eight different datasets including outgroup. 1d = 1 date (Perissodactyla); 2d = 2 dates (Perissodactyla and Plains zebra). B: Results are with a 25% burn-in for 3 different datasets without outgroup. 1d = 1 date (Plains zebra); n = no outgroup. (PDF) [file pone.0055950.s012.pdf]

Table S9a: Mean node ages estimated from BEAST. Results are with a 25% burn-in for eight different data sets including outgroups. 4p = 4 partitions; 5p = 5 partitions; BD = Births and Deaths; Y = Yule model; 1d = 1 date (Perissodactyla); 2d = 2 dates (Perissodactyla and Plains zebra); Sum. = Sumatran rhino; Woolly = woolly rhino; NWSLH = New World stilt-legged horse; Asses= *E. hemionus* and *E. kiang*; Zeb. = zebras; Donkey = *E. africanus* and *E. asinus*; Don. = Donkey. Total number of states is measured in millions of states (M). Node letter in parentheses corresponds to Figure 1.

Node	4pBD1d	4pBD2d	4pY1d	4pY2d	5pBD1d	5pBD2d	5Y1d	5Y2d	Average
Root	5.46E+07	5.43E+07	5.47E+07	5.38E+07	5.46E+07	5.41E+07	5.47E+07	5.36E+07	5.43E+07
Plains zebra	9.01E+05	7.07E+05	1.98E+06	7.26E+05	9.99E+05	7.14E+05	1.72E+06	7.34E+05	1.06E+06
Grevy's+Plains (B)	2.17E+06	1.90E+06	4.49E+06	2.78E+06	2.38E+06	1.99E+06	4.01E+06	2.68E+06	2.80E+06
Grevy's zebra	5.54E+05	5.06E+05	1.21E+06	7.52E+05	6.17E+05	5.37E+05	1.02E+06	7.30E+05	7.41E+05
Horses (J)	4.40E+05	4.01E+05	1.04E+06	6.31E+05	5.35E+05	4.70E+05	9.27E+05	6.55E+05	6.37E+05
Horse+NWSLH	4.70E+06	4.30E+06	8.88E+06	6.35E+06	4.95E+06	4.34E+06	7.93E+06	5.80E+06	5.91E+06
Mountain zebra	6.27E+05	5.68E+05	1.54E+06	9.19E+05	6.96E+05	6.13E+05	1.25E+06	8.75E+05	8.86E+05
Rhino+Tapir	4.37E+07	4.27E+07	4.63E+07	4.23E+07	4.50E+07	4.25E+07	4.68E+07	4.25E+07	4.40E+07
Rhinos	1.86E+07	1.74E+07	2.59E+07	2.11E+07	1.97E+07	1.73E+07	2.57E+07	2.07E+07	2.08E+07
Sum.+Woolly	9.22E+06	8.53E+06	1.25E+07	1.04E+07	9.53E+06	8.38E+06	1.24E+07	9.99E+06	1.01E+07
Donkey (H)	1.00E+06	9.12E+05	2.21E+06	1.48E+06	1.12E+00	9.72E+05	1.92E+06	1.40E+06	1.24E+06
Equids	7.39E+06	6.72E+06	1.47E+07	1.01E+07	7.71E+06	6.73E+06	1.26E+07	9.08E+06	9.38E+06
Non-caballine	4.63E+06	4.13E+06	9.45E+06	6.35E+06	4.81E+06	4.15E+06	8.05E+06	5.73E+06	5.91E+06
Kiang (E)	5.02E+05	4.57E+05	1.14E+06	7.28E+05	5.54E+05	4.85E+05	9.61E+05	6.97E+05	6.91E+05
Kulan/onager (G)	7.94E+05	7.20E+05	1.76E+06	1.15E+06	9.14E+05	8.00E+05	1.56E+06	1.16E+06	1.11E+06
Asses (F)	8.98E+05	8.10E+05	2.05E+06	1.30E+06	9.97E+05	8.74E+05	1.71E+06	1.26E+06	1.24E+06
Sussemione	4.87E+06	4.15E+06	9.43E+06	6.36E+06	4.79E+06	3.71E+06	8.51E+06	9.26E+06	6.39E+06
Donkey+Ass (D)	4.21E+06	3.69E+06	8.09E+06	5.60E+06	4.31E+06	3.29E+06	7.52E+06	6.63E+06	5.42E+06
Zeb+Don+Ass (C)	4.68E+06	4.04E+06	9.01E+06	6.15E+06	4.67E+06	3.59E+06	8.27E+06	6.94E+06	5.92E+06
Zebras (A)	3.73E+06	3.32E+06	7.45E+06	5.01E+06	3.91E+06	3.36E+06	6.49E+06	4.61E+06	4.74E+06
States	255M	256M	398M	265M	269M	268M	265M	262M	

Table S9b: Mean node ages estimated from BEAST. Results are with a 25% burn-in for 3 different data sets without outgroups. 4p = 4 partitions; 5p = 5 partitions; BD = Births and Deaths; Y = Yule model; 1d = 1 date (Plains zebra); n = no outgroup. Total number of states is measured in millions of states (M). NWSLH = New World stilt-legged horse; Asses = *E. hemionus* and *E. kiang*; Ass = Asses; Zeb. = zebras; Don. = Donkey; Donkey = *E. africanus* and *E. asinus*. Node letter in parentheses corresponds to Figure 1.

Node	4pBDn1d	4pYn1d	5pYn1d	Average
Plains zebra	6.86E+05	6.86E+05	6.85E+05	6.86E+05
Grevy's+Plains (B)	1.51E+06	1.42E+06	1.47E+06	1.46E+06
Grevy's zebra	3.83E+05	3.95E+05	4.02E+05	3.93E+05
Horses (J)	3.32E+05	3.68E+05	3.91E+05	3.64E+05
Horse+NWSLH	2.95E+06	2.38E+06	2.44E+06	2.59E+06
Mountain zebra	4.64E+05	5.01E+05	4.89E+05	4.84E+05
Donkey (H)	7.08E+05	7.05E+05	7.24E+05	7.13E+05
Equids	4.81E+06	3.96E+06	4.04E+06	4.27E+06
Non-caballine	3.14E+06	2.81E+06	2.83E+06	2.92E+06
Kiang (E)	3.53E+05	3.81E+05	3.74E+05	3.69E+05
Kulan/onager (G)	5.64E+05	5.96E+05	6.23E+05	5.94E+05
Asses (F)	6.45E+05	6.89E+05	6.83E+05	6.72E+05
Sussemione	3.13E+06	2.79E+06	2.83E+06	2.92E+06
Donkey+Ass (D)	2.83E+06	2.48E+06	2.55E+06	2.62E+06
Zeb+Don+Ass (C)	3.08E+06	2.75E+06	2.78E+06	2.87E+06
Zebras (A)	2.53E+06	2.25E+06	4.89E+05	1.75E+06
States	500M	500M	500M	
